# A Unified Platform for FCS and RICS Analysis with Advanced Statistical Inference

**DOI:** 10.1021/acsomega.5c12269

**Published:** 2026-03-16

**Authors:** Hamed Karimi, Otto Gustavson, Irina Česnokova, Jelena Branovets, Rikke Birkedal, Martin Laasmaa, Marko Vendelin

**Affiliations:** Laboratory of Systems Biology, Department of Cybernetics, 54561 Tallinn University of Technology, Akadeemia tee 15, Tallinn 12618, Estonia

## Abstract

Fluorescence correlation spectroscopy (FCS) and raster image correlation spectroscopy (RICS) are powerful techniques for measuring molecular diffusion, concentration, and dynamics in biological systems, yet current analysis tools lack unified frameworks that combine advanced statistical methods with high-performance computing. We present an open-source Python platform, IOCBIO FCS, that integrates FCS and RICS analysis with GPU-accelerated autocorrelation function calculation, robust statistical inference, and realistic optical modeling. The platform uniquely provides capabilities absent from existing open-source tools: direct incorporation of experimentally measured 3D point spread functions into fitting procedures, comprehensive statistical frameworks encompassing Bayesian inference alongside generalized, weighted, and ordinary least-squares methods for rigorous uncertainty quantification, and combined multiple-angle RICS analysis for characterizing anisotropic diffusion in complex biological systems. Additional features include image partitioning for spatial parameter mapping, advanced filtering strategies for data quality control, and comprehensive visualization of fitted results, residuals, posterior distributions, and parameter maps. This platform establishes a reproducible workflow bridging modern fluorescence microscopy with quantitative analysis of molecular transport across biophysics, biochemistry, and cell biology research.

## Graphical abstract

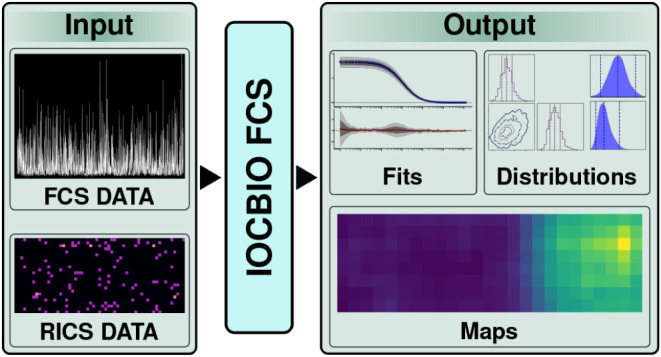

## Introduction

The quantitative characterization of molecular transport phenomena and intermolecular interactions represents a fundamental challenge in modern biophysics, biochemistry, and cellular biology. Understanding how molecules move, interact, and organize within complex biological environments is essential for elucidating cellular processes ranging from signal transduction and enzymatic reactions to membrane dynamics and organelle function.^1–4^ For example, in specialized cells such as cardiomyocytes (CMs), diffusion is significantly restricted, impacting intracellular energy transfer and signaling.^5–15^

Advanced fluorescence-based correlation techniques have emerged as powerful methodological approaches to address these questions, providing unprecedented insights into molecular behavior at physiologically relevant concentrations and under cellular conditions.

Fluorescence correlation spectroscopy (FCS) and raster image correlation spectroscopy (RICS) have established themselves as indispensable tools for quantifying molecular dynamics in living and solution-based systems.^16–20^ FCS exploits the inherent fluorescence intensity fluctuations arising from molecular motion and interactions within an observation volume, enabling precise determination of diffusion coefficients, molecular concentrations, and binding kinetics with single-molecule sensitivity. Complementarily, RICS extends correlation analysis to scanning fluorescence microscopy by analyzing intensity fluctuations across raster-scanned images, thereby providing spatially resolved measurements of molecular transport properties.

The biological significance of these techniques is evidenced by their widespread application across diverse research domains. In membrane biology, FCS and RICS have revealed the complex dynamics of lipid rafts, membrane protein clustering, and receptor–ligand interactions, providing crucial insights into cellular signaling mechanisms.^21–24^ In cellular transport studies, these methods have elucidated anomalous diffusion phenomena in the cytoplasm and nucleus, revealing how macromolecular crowding, active transport processes, and intracellular barriers influence molecular mobility.^25–28^ Furthermore, in biochemical research, correlation spectroscopy has proven invaluable for studying protein folding dynamics, enzyme–substrate interactions, and drug-target binding kinetics both in vitro and in living cells.^29,30^

Despite their widespread adoption and proven utility, the analysis of FCS and RICS data presents substantial computational and methodological challenges that limit their accessibility and reliability.^31,32^ Raw fluorescence signals are inherently susceptible to experimental artifacts including detector noise, photobleaching, photoblinking, and mechanical drift, all of which can significantly distort correlation functions and compromise parameter extraction.^33–36^ The presence of immobile fractions, heterogeneous populations, or anomalous diffusion behaviors further complicates data interpretation and requires sophisticated analytical approaches. Additionally, biological systems frequently exhibit spatial heterogeneity and anisotropic transport properties that necessitate advanced correlation analysis methods capable of resolving directional dependencies and local variations in molecular dynamics.^27,37^

Traditional fitting approaches based on least-squares optimization are relatively fast but often fail to provide reliable uncertainty estimates and may converge to local minima, especially when dealing with noisy data or complex multicomponent systems.^38^ By contrast, Bayesian inference frameworks for parameter estimation provide superior uncertainty quantification and model selection capabilities, enabling more rigorous and interpretable analysis of correlation data.^39–41^

The software landscape for FCS and RICS remains limited. Although RICS analyses incorporating variable scanning speeds and angles have been previously implemented^27,37^ to date, no open-source software offering these analytical capabilities has been made publicly accessible. Other existing open-source tools are restricted to either FCS or RICS analysis, often rely exclusively on CPU-based computation for autocorrelation function (ACF) calculation, omit modern statistical approaches for robust uncertainty quantification, lack support for Bayesian inference, and do not allow fitting with experimentally measured 3D point spread functions (PSFs).^42–50^

To overcome these limitations, the present work introduces a unified, open-source platform that integrates several critical capabilities into a reproducible and statistically rigorous workflow for molecular transport analysis: 1—visualization tools for quality control, data exploration, and data filtering before ACF calculation; 2—flexible ACF computation with support for both CPU and GPU acceleration, and multiple correlation modes optimized for different experimental geometries, with additional functionality for image splitting to enable parameter mapping and support for laser scanning at arbitrary angles and speeds; 3—advanced data filtering strategies after ACF calculation, including pre-analysis of diffusion coefficient and concentration for systematic exclusion of unreliable measurements prior to fitting; 4—flexible diffusion modeling supporting both experimentally measured 3D PSFs and analytical 3D Gaussian ellipsoid approximations; 5—robust fitting frameworks incorporating least-squares methods alongside Bayesian inference with multiple noise models; 6—comprehensive visualization of fitted results with support for parameter mapping, residual analysis, and posterior distribution visualization for estimated parameters.

The primary methodological novelty of the present platform lies in its unified open-source implementation that, for the first time, combines RICS analysis with arbitrary scan angles and speeds, Bayesian inference with support for generalized least-squares (GLS) to account for correlated errors in parameter estimation and uncertainty quantification, fitting with experimentally measured 3D PSFs, and an advanced filtering strategy based on diffusion-concentration pre-analysis within a single reproducible workflow supporting both FCS and RICS. The ability to incorporate experimentally measured PSFs and to support RICS acquisition at arbitrary scan angles is particularly important in anisotropic systems such as cardiomyocytes, where molecular transport is spatially constrained and deviations from idealized PSF geometry can significantly bias extracted diffusion coefficients. Additionally, the platform incorporates GPU-accelerated ACF computation, which significantly reduces processing time for large data sets while maintaining full compatibility with CPU-based operation. By bringing these capabilities together in a statistically rigorous and computationally scalable framework, the platform enables new biological insights into spatially heterogeneous and anisotropic molecular dynamics that were previously difficult to obtain reliably with existing tools.

## Methods

### Overview

The Methods section outlines the implementation of the software framework for FCS and RICS analysis. The workflow comprises three major components: calculation of ACFs; fitting of ACFs with different models that support both Gaussian and experimentally measured PSFs, implemented within frequentist and Bayesian inference frameworks; and visualization of data across all stages of analysis. Experimental measurements were performed using solution measurements as well as live and fixed cell preparations where mitochondria were fluorescently labeled. The software was developed in Python with a command line interface, relying on widely used scientific packages for numerical computation, data management, and visualization. This enables scriptable, reproducible workflows and deployment on computing clusters where graphical interfaces are impractical. For some tasks, interactive cursor-based menus support parameter filtering (scanning angles, speeds) and RICS spatial map visualization. This hybrid approach balances automation with interactivity.

### ACF Calculation

The software computes the ACF to quantify fluctuations in fluorescence intensity in both FCS and RICS data sets. For FCS, the ACF is calculated as a function of lag time (τ), capturing correlations of the fluorescence intensity *I*(*t*) according to

1 $$G\left(\tau \right)=\frac{\left\langle \delta I\left(t\right)\delta I\left(t+\tau \right)\right\rangle }{\langle I\rangle^{2}}$$

where δ*I*(*t*) = *I*(*t*) – ⟨*I*⟩ represents deviations from the mean. The correlation is normalized by the square of the mean photon count, producing a dimensionless function suitable for quantitative analysis.^16,17^

For RICS, the software computes a spatial ACF in which temporal information is intrinsically encoded by the scanning pattern. The spatial ACF is defined as

2 $$G\left(\xi ,\psi \right)=\frac{\langle \delta I\left(x,y\right)\delta I\left(x+\xi ,y+\psi \right)\rangle_{x,y}}{\langle I\left(x,y\right)\rangle_{x,y}^{2}},,\mathrm{with}\;\delta I\left(x,y\right)=I\left(x,y\right)-\langle I\left(x,y\right)\rangle_{x,y}$$

Here, ξ and ψ denote pixel displacements (spatial lags) along the fast- and slow-scan axes, respectively, and ⟨·⟩_*x,y*_ indicates averaging over all spatial locations in both *x* and *y* directions.

Temporal information is encoded in the scanning process: a displacement of ξ pixels along the *x*-axis corresponds to an effective delay of ξ*t*_pix_, while a displacement of ψ lines along the *y*-axis corresponds to ψ*t*_line_. Thus, the effective lag time is

3 $$\tau \left(\xi ,\psi \right)=\xi t_{\mathrm{pix}}+\psi t_{\mathrm{line}}$$

Although the ACF in RICS is expressed in spatial coordinates, each spatial lag can be mapped to an effective temporal lag.^19^

For RICS data sets acquired in structured environments such as cells, the user can choose to subtract the background to remove nonspecific fluorescence and stationary contributions before normalization. In this case, images are grouped by acquisition conditions, and an average image for each group is subtracted from individual frames. The resulting arrays are then normalized by dividing by the squared variance of the background-subtracted signal. In cases such as diffusion in solution, where background is negligible, each image is normalized by subtracting its mean and dividing by the squared mean intensity. These normalization procedures yield a robust, dimensionless measure of correlation that is suitable for extraction of diffusion and dynamic parameters across different experimental conditions.

For RICS data sets, the software implements three configurations for spatially resolved autocorrelation analysis via image splitting. The image series is divided into user-defined sectors along the horizontal and vertical axes, ensuring that all sectors are fully contained within the image and evenly distributed across the imaged area. Sector centers are determined based on these restrictions, and Figure 1 illustrates the three available configurations. In the first configuration, sector self-correlation, the ACF is computed for each sector with itself, enabling direct mapping of local heterogeneities when photon counts are sufficient (Figure 1A). In the second configuration, larger-area self-correlation, a larger area centered on each sector is correlated with itself, thereby increasing the sampling volume and improving statistical averaging (Figure 1B). This approach is particularly useful when photon counts are limited.^51^ In the third configuration, pair-correlation, a smaller reference area is correlated with a larger concentric area, providing sensitivity to directional transport and spatial connectivity between different scales (Figure 1C). Together, these modes enable multiscale mapping of molecular dynamics, from local fluctuations to anisotropic or active transport processes within cells.

**Figure 1:**
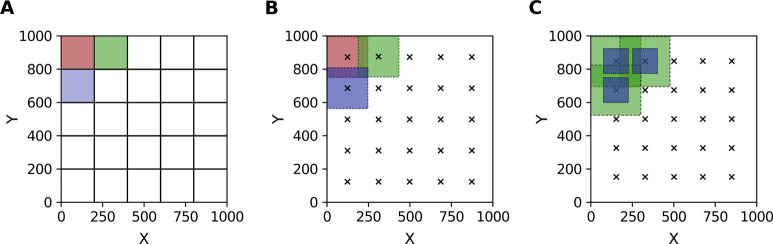
Image-splitting configurations for spatially resolved autocorrelation analysis in RICS data sets (1000 × 1000 pixels). (**A**) Sector self-correlation, where the autocorrelation function (ACF) is computed for each sector with itself. Colored squares indicate example correlation areas. (**B**) Larger-area self-correlation, where the ACF is computed for each larger area with itself. Colored squares indicate example correlation areas, and cross symbols mark sector centers. (**C**) Pair-correlation between smaller and larger areas (blue and green squares, respectively). Cross symbols mark sector centers.

### Diffusion Models

The theoretical fit of the ACF relies on the PSF of the microscope. The PSF is handled in three different ways: first, the experimentally measured PSF can be directly^52^ used in the integrals of the correlation model (numerical evaluation, computationally slower), second, the experimental PSF can be reduced to an equivalent Gaussian ellipsoid by extracting the waist parameters ω_*x*_, ω_*y*_, and ω_*z*_, which are then used in the analytical formulas, or third, the user may directly set ω_*x*_, ω_*y*_, and ω_*z*_ to desired values. For all three options, an additional PSF-scale factor can be applied in the range 0–2 (default value 1), allowing further flexibility in fitting.

The 3D Gaussian ellipsoid approximation of the PSF is given by^17,53^

4 $$\mathrm{PSF}\left(x,y,z\right)=\exp\left(-\frac{2x^{2}}{\omega_{x}^{2}}-\frac{2y^{2}}{\omega_{y}^{2}}-\frac{2z^{2}}{\omega_{z}^{2}}\right)$$

The effective focal volume is defined by^17,54^

5 $$V_{\mathrm{eff}}=\frac{{[\int \mathrm{PSF}(r)dV]}^{2}}{\int {\mathrm{PSF}}^{2}\left(r\right)dV}$$

and is used to determine the molecular occupancy within the observation region. Here, ***r*** = (*x*, *y*, *z*) denotes the spatial coordinates in the focal volume *V*.

For RICS, the autocorrelation depends both on the spatial lag (pixel displacements mapped into sample coordinates) and on the lag time introduced by the raster scan. In the anisotropic Gaussian PSF approximation, the spatiotemporal ACF for a displacement **Δ** = (Δ*x*, Δ*y*, Δ*z*) and lag time τ can be written as,^19,20,27^

6 $$G\left(\Delta ,\tau \right)=\frac{1}{V_{\mathrm{eff}}\left\langle C\right\rangle }\underset{i\in \left\{x,y,z\right\}}{\prod }\left[\frac{1}{\sqrt{\pi \left(4DC_{i}\tau +\omega_{i}^{2}\right)}}\exp\left(-\frac{\Delta_{i}^{2}}{4DC_{i}\tau +\omega_{i}^{2}}\right)\right]$$

where ⟨*C*⟩ is the mean concentration, *DC*_*i*_ the diffusion coefficient along axis *i* ∈ {*x*, *y*, *z*}, and ω_*i*_ the PSF width along axis *i*. The product runs over the three spatial axes to account for anisotropic diffusion and the 3D PSF. In this formulation, diffusion is direction dependent with *DC*_*x*_, *DC*_*y*_, and *DC*_*z*_. Because RICS samples spatial lags only in the imaging plane (Δ*x*, Δ*y*) while Δ*z* = 0, the acquisition geometry does not impose any equality between *DC*_*z*_ and the in-plane coefficients. Axial diffusion contributes via the 3D PSF to the temporal evolution of the ACF, and *DC*_*z*_ may equal *DC*_*x*_ or *DC*_*y*_ only under sample-specific assumptions (e.g., isotropic 3D: *DC*_*x*_ = *DC*_*y*_ = *DC*_*z*_; quasi-2D: *DC*_*z*_ = 0; uniaxial anisotropy: *DC*_*x*_ ≠ *DC*_*y*_ = *DC*_*z*_). A two-component anisotropic extension is obtained by a linear mixture of two component contributions (fraction *f* for component 1):

7 $$G\left(\Delta ,\tau \right)=\frac{1}{V_{\mathrm{eff}}\left\langle C\right\rangle }\left[f\underset{i\in \left\{x,y,z\right\}}{\prod }\frac{1}{\sqrt{\pi \left(4DC_{1,i}\tau +\omega_{i}^{2}\right)}}\exp\left(-\frac{\Delta_{i}^{2}}{4DC_{1,i}\tau +\omega_{i}^{2}}\right)+\left(1-f\right)\underset{i\in \left\{x,y,z\right\}}{\prod }\frac{1}{\sqrt{\pi \left(4DC_{2,i}\tau +\omega_{i}^{2}\right)}}\exp\left(-\frac{\Delta_{i}^{2}}{4DC_{2,i}\tau +\omega_{i}^{2}}\right)\right]$$

with *DC*_1,*i*_ and *DC*_2,*i*_ the axis-dependent diffusion coefficients of components 1 and 2, respectively (the isotropic two-component form is recovered when *DC*_1,*i*_≡*DC*_1_ and *DC*_2,*i*_≡*DC*_2_).

To account for fluorophore triplet dynamics, the correlation function is multiplied by^17^

8 $$\left(1+\frac{\mathrm{TSA}}{1-\mathrm{TSA}}\exp\left(-\frac{\tau }{\tau_{T}}\right)\right)$$

where *TSA* denotes the triplet-state amplitude and τ_*T*_ the triplet-state relaxation time.

The sample-frame displacements Δ*x*, Δ*y*, Δ*z* are obtained from pixel displacements (ξ, ψ, ζ) and the pixel sizes by a rotation by the scan angle α:

9 $$\left[\begin{array}{c} \Delta x\\ \Delta y\\ \Delta z \end{array}\right]=\left[\begin{array}{ccc} \cos\alpha & -\sin\alpha & 0\\ \sin\alpha & \cos\alpha & 0\\ 0 & 0 & 1 \end{array}\right]\cdot \left[\begin{array}{c} \xi s_{\xi }\\ \psi s_{\psi }\\ \zeta s_{\zeta } \end{array}\right]$$

where *s*_ξ_, *s*_ψ_, *s*_ζ_ denote the pixel sizes along the corresponding axes in physical units.

The correlation lag time in RICS τ­(ξ, ψ) is determined by the scanning process, with τ­(ξ, ψ) = ξ*t*_pix_ + ψ*t*_line_ as defined above. The pixel sizes *s*_ξ_, *s*_ψ_, *s*_ζ_ are instead used to relate spatial displacements to the physical dimensions of the PSF.

Evaluating eq 6 (or eq 7) at zero spatial lag, Δ*x* = Δ*y* = Δ*z* = 0, removes the exponential spatial terms and reduces the expression to the purely temporal ACF used in point FCS. Thus, FCS corresponds to the special case of the RICS formulation with vanishing spatial lag and direct specification of the temporal lag τ. Extensions to two-component diffusion or the inclusion of triplet dynamics can be incorporated.

For the experimentally measured PSF, the same diffusion equations as in^27^ are applied for the different models.

### Fitting Frameworks

The software implements six complementary parameter estimation approaches by combining two statistical frameworks—frequentist and Bayesian inference—with three error treatment methods.

The frequentist framework estimates parameters by minimizing the chi-squared statistic

10 $$\chi^{2}\left(\theta \right)={[y-f(x,\theta )]}^{T}W\left[y-f\left(x,\theta \right)\right]$$

where *y* represents the observed data, *f*(*x*, θ) is the model function, θ are the parameters to be estimated, and *W* is the weight matrix determined by the error structure.

For ordinary least-squares (OLS) with uniform errors, the optimization problem reduces to minimizing the sum of squared residuals $S\left(\theta \right)=\overset{n}{\underset{i=1}{\sum }}{[y_{i}-f(x_{i},\theta )]}^{2}$ , which is equivalent to using *W* = *I* (identity matrix) in the chi-squared formulation. The common error variance is subsequently estimated from the optimal residuals as ${\mathrm{\sigma ̂}}^{2}=\frac{1}{n-p}S\left(\mathrm{\theta ̂}\right)$ , where θ̂ are the fitted parameters, *n* is the number of data points, and *p* is the number of parameters. This approach assumes uniform weighting across all data points. When measurement uncertainties vary across data points, weighted least-squares (WLS) employs $W=\mathrm{diag}\left(\frac{1}{\sigma_{1}^{2}},\frac{1}{\sigma_{2}^{2}},...,\frac{1}{\sigma_{n}^{2}}\right)$ , providing optimal parameter estimates under heteroscedastic conditions. For correlated measurement errors with known covariance matrix Σ, generalized least-squares (GLS) uses *W* = Σ^–1^ to account for both heteroscedasticity and correlation structure, ensuring efficient parameter estimation when errors exhibit complex dependencies.^39,40,55–57^

The covariance matrix Σ for FCS is empirically estimated from *N* independent traces acquired under identical experimental conditions. Each trace *k* yields an ACF, and the sample covariance matrix is computed as

11 $$\Sigma_{i,j}=\frac{1}{N-1}\overset{N}{\underset{k=1}{\sum }}\left[G_{k}\left(\tau_{i}\right)-\mathrm{G̅}\left(\tau_{i}\right)\right]\left[G_{k}\left(\tau_{j}\right)-\mathrm{G̅}\left(\tau_{j}\right)\right]$$

where *G*_*k*_(τ_*i*_) is the ACF value at lag time τ_*i*_ for trace *k* and $\mathrm{G̅}\left(\tau_{i}\right)=\frac{1}{N}\overset{N}{\underset{k=1}{\sum }}G_{k}\left(\tau_{i}\right)$ is the mean ACF. Diagonal elements Σ_*i,i*_ quantify variance at each lag time, while off-diagonal elements Σ_*i,j*_ (*i* ≠ *j*) capture correlations between different lag times. These correlations arise from temporal overlap in the correlation calculation and photon detection statistics. Reliable covariance estimation requires *N* to be substantially larger than the number of ACF points to ensure matrix stability.^40^

The Bayesian framework treats parameters as random variables and characterizes their posterior distribution through *p*(θ|*y*) ∝ *p*(*y*|θ)*p*(θ), where *p*(*y*|θ) is the likelihood function and *p*(θ) represents prior information. For uniform errors, the likelihood function assumes Gaussian distributions

12 $$p\left(y|\theta ,\sigma \right)=\overset{n}{\underset{i=1}{\prod }}\frac{1}{\sqrt{2\pi \sigma^{2}}}\exp\left(-\frac{{[y_{i}-f(x_{i},\theta )]}^{2}}{2\sigma^{2}}\right)$$

When individual error estimates are available, the heteroscedastic likelihood incorporates these uncertainties

13 $$p\left(y|\theta \right)=\overset{n}{\underset{i=1}{\prod }}\frac{1}{\sqrt{2\pi \sigma_{i}^{2}}}\exp\left(-\frac{{[y_{i}-f(x_{i},\theta )]}^{2}}{2\sigma_{i}^{2}}\right)$$

For correlated errors, the likelihood utilizes the full covariance structure^40^

14 $$p\left(y|\theta \right)=\frac{1}{\sqrt{{\left(2\pi \right)}^{n}\left|\Sigma \right|}}\exp\left(-\frac{1}{2}{[y-f(x,\theta )]}^{T}\Sigma^{-1}\left[y-f\left(x,\theta \right)\right]\right)$$

In this expression, *y* – *f*(*x*, θ) represents the residual vector containing all *n* data points. The covariance matrix Σ accounts for correlations among these residuals, Σ^–1^ denotes its inverse, and |Σ| its determinant, which ensures proper normalization of the multivariate Gaussian likelihood.

In the Bayesian inference framework implemented in our software, this likelihood is combined with prior distributions to compute the posterior probability of the model parameters. The Bayesian evidence (*Z*) quantifies the marginal likelihood obtained by integrating the likelihood over the parameter space and serves as a basis for objective model comparison, naturally implementing Occam’s razor.^58–60^

### Confocal Microscopy System

Experiments were conducted on a custom-built confocal microscope,^27^ equipped with a water-immersion 60× objective lens (UPLSAPO; NA 1.2; Olympus). For FCS and RICS measurements, Alexa Fluor 647 fluorophores were excited using a 633 nm laser source (05-LHP-151, Melles Griot, US) focused onto the sample plane. Dye was selected to minimize impact of autofluorescence in cardiomyocytes which is significant at shorter wavelengths.

Fluorescence signals were collected through a long-pass filter (F76-631, Semrock, Rochester, NY) and detected using an avalanche photodiode detector (SPCMAQRH-54, Excelitas Technologies, Pittsburgh, PA), using a PCIe data acquisition board with temporal sampling of 1 and 3 μs (PCIe-6353, National Instruments, Austin, TX). For mitochondrial imaging experiments, MitoTracker Green-labeled cells were excited using a 488 nm laser (0488L11A-NI-NT-NF, Integrated Optics UAB, Lithuania), and fluorescence was captured through a bandpass filter (550/88 nm; FF01-550/88-25, Semrock, Rochester, NY) with 30 μs pixel time.

### Solution Experiments

Experiments were performed using filtered Alexa Fluor 647 Dextran 10K in water or a glycerol/water mixture at room temperature (22°C). Samples were allowed to equilibrate for 10 min to ensure sedimentation and stability before measurements. A temporal sampling of 1 μs was used for all experiments, unless explicitly stated otherwise.

### Cardiomyocyte Experiments

All animal studies were conducted in compliance with Directive 2010/63/EU of the European Parliament regarding the protection of laboratory animals and received approval from the Project Authorisation Committee for Animal Experiments of the Estonian Ministry of Rural Affairs.

For live cell experiments, ventricular CMs were freshly isolated from female Wistar Han rats (61 days old; Envigo RMS, 5961 NM Horst, The Netherlands) for determining diffusion coefficients. Cell isolation followed the protocol described earlier;^61^ wash solution composition described in ref 62. CMs were subsequently transferred to a reusable silicone chamber (94.6077.434, flexiPERM, SARSTEDT AG & Co. KG, Nümbrecht, Germany) mounted on a coverslip and bathed in an intracellular-like medium. This solution comprised 0.5 mM EGTA, 3.0 mM KH_2_PO_4_, 3.0 mM MgCl_2_, 20 mM Hepes, 110 mM sucrose, 20 mM taurine, 0.5 mM dithiothreitol, 60 mM lactobionate, 5 mM glutamate, 2 mM malate, 5.0 mM MgATP, 10 mM PCr, and 10 nM Alexa Fluor 647 Dextran 10K, supplemented with bovine serum albumin (5 mg/mL) and adjusted to pH 7.1 at 25°C using KOH.

For fixed cell experiments, ventricular CMs were obtained from a male mouse (221 days old) with arginine-glycine amidinotransferase (AGAT) heterozygosity on a pure C57BL/6J genetic background.^62,63^ Isolation procedure is described in ref 62. Freshly isolated CMs were fixed in 4% paraformaldehyde (PFA) for 10 min, washed and stored in PBS at 4°C until further use. Fixed CMs were transferred to a reusable silicone chamber, mounted on a coverslip coated with Cell-Tak cell adhesive (354240, Corning, US), and bathed in a solution containing PBS and 50 nM Alexa Fluor 647 Dextran 10K.

For both live and fixed experiments, prior to measurements, CMs were incubated with MitoTracker Green FM (M7514, Invitrogen, Eugene, OR) at 250 nM for 10 min to visualize mitochondrial networks. Cell membrane permeabilization was achieved using a glass micropipette controlled by a precision micromanipulator (SMXS-K-L-EUR, Sensapex, Oulu, Finland). The micropipettes, featuring tip diameters of 0.5 μm, were fabricated from 1.0 mm glass capillaries (TW100F-3, World Precision Instruments, Sarasota, US) using a micropipette puller (PC-10, Narishige, Japan). After approximately 5 min to allow cell adherence to the coverslip within the silicone chamber, individual cells were selected for analysis. After membrane permeabilization, cells were allowed to equilibrate with the external medium for 5 min. Confocal imaging of the mitochondrial architecture was first performed, followed by selection of discrete square regions within the cell for subsequent diffusion analysis.

### PSF Characterization

PSF measurements were performed using TetraSpeck microsphere test slides prepared according to the protocol described in ref 64. The stock suspension (T7279, Invitrogen, Eugene, OR) was diluted 1:10,000 in distilled water, and a droplet of the diluted solution was deposited onto a 0.17 mm coverslip and dried at room temperature. After drying, the sample was mounted with immersion oil (Carl Zeiss Immersol W, Oberkochen, Germany; refractive index *n* = 1.334 at 23°C) and sealed with a glass slide. Images were acquired in regions containing well-separated microspheres to avoid overlapping Airy patterns near the focal plane. The acquisition settings included lateral pixel sizes below 40 nm and axial step sizes of 100 nm. Multiple 3D stacks were collected for subsequent analysis. Individual microspheres were localized within each stack and aligned based on centroid positions obtained by least-squares optimization (eq 4). The averaged point source profile was then fitted with eq 4 to extract ω_*x*_, ω_*y*_, and ω_*z*_, used in the correlation analysis.

### Synthetic Trace

Synthetic fluorescence traces were generated using the simulation package provided in FITSA.^65^ For single-trace FCS, 100 particles freely diffusing in a 2.5 × 2.5 × 7 μm^3^ box (*x*, *y*, *z*) with diffusion coefficient *DC* = 100 μm^2^ s^–1^ were simulated over 80 s with a time step of 1 μs. For multitrace FCS, 25 particles in a larger 8 × 8 × 12 μm^3^ box with *DC* = 20 μm^2^ s^–1^ were simulated to produce 2000 traces, each 30 s long with the same time step. In both cases, a symmetric Gaussian PSF with lateral waist ω_*xy*_ = 0.3 and axial waist ω_*z*_ = 1.1 μm was used consistently for data generation and model fitting.

### Benchmarking

Computational performance benchmarking was evaluated using Linux PC with AMD Ryzen 9 9950X CPU, NVIDIA GeForce RTX 4080 SUPER with 16 GB GDDR6X memory GPU, 128 GB DDR5 RAM, Debian GNU/Linux 12, Python 3.11.2, NumPy 2.4.1, SciPy 1.16.3, CuPy 13.6.0, Cython 3.2.4, CUDA Toolkit 13.1, NVIDIA Driver 590.48.01.

GPU memory requirements for ACF calculation scale differently for FCS and RICS protocols. For FCS, memory consumption is ∼80 bytes per time point (e.g., 60-million-point trace, equivalent to 1 min acquisition at 1 μs time resolution, requires ∼4.8 GB). For RICS, memory consumption is ∼120 bytes per pixel for single-image analysis (higher than FCS due to 2D array handling and sector processing overhead; e.g., 1000 × 1000 images require ∼120 MB), and ∼320 bytes per pixel for 30-image background slots (e.g., 30 images of 1000 × 1000 require ∼320 MB). The sector splitting does not increase memory requirements as sectors are processed sequentially. For RICS experiments, GPU memory requirements are determined by the number of images per slot (when background subtraction is enabled) rather than the total number of images in the experiment, as images and slots are processed sequentially.

Performance times correspond to elapsed real time measured under the hardware and software conditions described above. Total runtime for complete analysis tasks (e.g., ACF calculation and full fitting workflows) was measured using bash shell time keyword. The duration of the model fitting stage was measured separately within the analysis code using Python time module, enabling separation of fixed overhead components (data loading and output writing) from fitting computation.

### Data Format

The software uses HDF5 as the standardized input format for ACF calculation, supporting both FCS and RICS data sets.^66^ After computation, the resulting ACFs are saved in HDF5 format, enabling seamless reloading for further analysis or visualization. The fitted ACF results are also stored in HDF5 format and can be reloaded for visualization of fitted curves, residuals, or parameter maps. For Bayesian inference, posterior samples can additionally be saved in NetCDF (nc) format following the ArviZ data storage standards.^67^

### Implementation

The software implements ACF calculations on both CPU and GPU, using NumPy for CPU-based processing and CuPy for GPU acceleration.^68–70^ GPU computation provides substantially faster analysis, particularly for RICS and multitrace FCS data sets. Integration over the experimentally measured PSF is accelerated with Cython, enabling efficient computation. Parameter estimation is supported under both frequentist and Bayesian frameworks. Nonlinear least-squares (NLS) optimization was performed using the SciPy library.^71^ Bayesian posterior sampling relied on nested sampling algorithms^72,73^ provided by UltraNest, which also computes the log-evidence (log *Z*) for model comparison.^60^ Posterior diagnostics, including parameter correlations, are displayed using corner plots implemented in the UltraNest package.^60^ Visualization is handled through Matplotlib figure window.

### Parameter Constraints and Prior Bounds

The software applies bounded priors to all fitted parameters in both NLS and Bayesian frameworks, constraining them to physically meaningful ranges with enforced non-negativity to prevent nonphysical solutions. By default, diffusion coefficients are constrained to 0–5000 μm^2^ s^–1^, concentrations to 0–1000 nM, triplet-state amplitudes to 0–1, triplet-state relaxation times to 0.01–10^4^ μs, and the PSF scaling factor to 0–2. For two-component diffusion models, identifiability constraints (e.g., *DC*_1_ < *DC*_2_) prevent label-switching during parameter estimation. All prior ranges can be adjusted by the user via command-line options or configuration files to reflect system-specific knowledge.

### Goodness-of-Fit Assessment

Goodness-of-fit is quantified using the chi-squared statistic evaluated at the fitted parameters, χ^2^(θ̂) (eq 10), and the reduced chi-squared

15 $$\chi_{\nu }^{2}=\chi^{2}\left(\mathrm{\theta ̂}\right)/\left(n-p\right)$$

where *n* is the number of data points and *p* the number of fitted parameters. The definition of χ^2^(θ̂) naturally depends on the assumed error model through the weight matrix *W* (OLS, WLS, or GLS; see *Fitting Frameworks*). The software reports the sum of squared raw residuals (SSR), the chi-squared statistic based on the specified error model, as well as the reduced chi-squared, which normalizes χ^2^(θ̂) by the degrees of freedom and provides a scale-independent measure of fit quality. For Bayesian inference, these metrics are computed using median posterior parameter samples. While these summary statistics provide overall fit quality measures, visual inspection of residual patterns as a function of lag time (or pixel displacement for RICS) remains essential for detecting systematic deviations. For Bayesian inference, posterior credible intervals (shown as shaded regions in figures) provide additional assessment of model adequacy.

## Results

The software is designed to analyze both FCS and RICS data, enabling studies of diffusion in cells and various solutions. The analysis integrates multiple capabilities, including visualization for data inspection, flexible model selection for diffusion and photophysical processes, robust fitting strategies, error modeling, and spatial parameter mapping. To demonstrate its performance and versatility, we applied the software to synthetic and experimental data sets using the full set of diffusion models described in the *Methods* section, with parameter estimation performed by both nonlinear least-squares (NLS) and Bayesian approaches under different error assumptions.

Figure 2 shows the complete analysis workflow, illustrating how the platform integrates FCS and RICS analyses within a unified, modular pipeline. Some of these features are described below and demonstrated in *Application Examples*.

**Figure 2:**
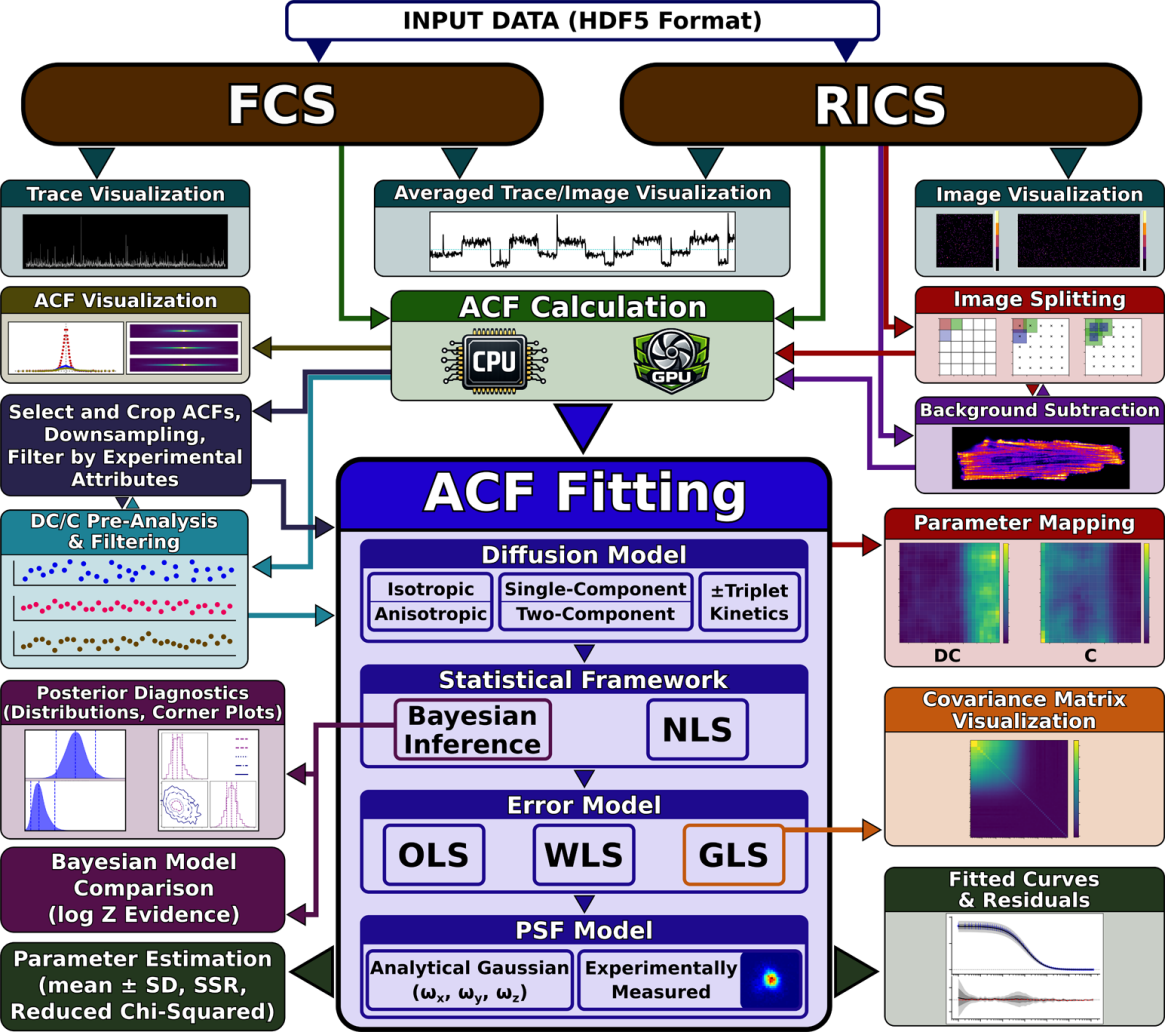
IOCBIO FCS analysis workflow. Schematic overview of the complete analysis pipeline from data input through results visualization. The platform accepts fluorescence correlation spectroscopy (FCS) photon time traces and raster image correlation spectroscopy (RICS) image stacks in HDF5 format, supporting both techniques within a unified framework. Quality control via trace/image visualization precedes GPU- or CPU-accelerated ACF calculation. Post-correlation processing includes flexible ACF selection and cropping, logarithmic downsampling, and filtering by experimental attributes (scanning angle, speed). *DC-* and *C*-based pre-analysis enables quality screening and filtering before main fitting. The ACF fitting module provides comprehensive model selection (diffusion type, statistical framework, error model, point spread function (PSF) treatment). Bayesian inference yields posterior diagnostics and model comparison via log-evidence, while both Bayesian and nonlinear least-squares (NLS) approaches produce fitted curves with residuals and parameter estimates. RICS-specific features include image splitting for spatial parameter mapping and background subtraction. Outputs include covariance matrix visualization for generalized least-squares (GLS), parameter maps, and comprehensive fit diagnostics.

### Software Features

#### Experimental Data Handling and ACF Calculation

The software supports both FCS and RICS with options for visualizing individual fluorescence traces, averaged traces across multiple measurements (FCS), single image frames, and averaged images from multiframe acquisitions (RICS). These modes enable detection of bleaching, drifts, illumination inhomogeneity, or sample motion, and together provide diagnostic tools for assessing measurement stability before correlation. Unstable traces or frames can then be excluded to ensure that only representative data contribute to the ACF.

The software computes the ACF for both FCS and RICS, with options that provide flexibility in trace/image selection. Prior to ACF calculation, images can be split into smaller regions to enable parameter mapping or grouped by acquisition conditions to enable background subtraction in RICS. For FCS, the photon count trace can be restricted to a chosen time interval to exclude unstable segments. After ACF computation, cropping options allow restriction to a defined τ-range (FCS) or truncation of the central portion of the 2D-ACF (RICS), retaining the region containing molecular dynamics information.

The software also provides multiple visualization modes for ACF inspection. Temporal ACFs can be plotted as a function of τ on linear or logarithmic axes for both FCS and RICS techniques. In RICS, temporal profiles can be extracted line by line from the 2D-ACF, with the option to select additional lines to probe spatial variations. The software also supports inspection of spatial correlations, where the ACF is plotted as a function of pixel shift (Δ*x*) across lines. Curves may be downsampled logarithmically to preserve detail at short delays while compressing longer delays. For global assessment, the 2D-ACF can be displayed as a heatmap, providing an overview of correlation amplitudes and anisotropy.

#### Data Filtering and Fitting

The software incorporates a multistage filtering strategy after ACF calculation. Users can select specific ACFs from FCS or RICS data sets without recalculating, and may exclude initial ACF points to remove contributions from detector shot noise and triplet-state kinetics. The fitting range can be further restricted to a defined number of ACF points per line or to selected lines in the spatial correlation (RICS), providing fine control over which portions of the correlation data are analyzed. Data sets can also be filtered by acquisition attributes such as scanning angle, speed, or other experiment-specific keys, ensuring that only measurements obtained under consistent conditions contribute to the final analysis.

In addition, the software can compute values of *DC* and *C* for each individual trace/image and visualizes them as scatter plots. This pre-analysis enables assessment of parameter distributions, identification of systematic trends, and exclusion of data with distorted *DC* or *C* values prior to the main fitting. When RICS frames are divided into smaller sectors, the number of ACFs increases, allowing finer-grained filtering. This is particularly useful for identifying problematic regions in samples with large, slow-moving particles; excluding only the affected sectors improves robustness and reduces bias in diffusion estimates. An early version of this software and filtering approach was previously applied to such large-particle filtering.^74^ This tiered workflow ensures that downstream fitting is based on reproducible, high-quality data while allowing users to tailor filtering strategies to experimental conditions.

For fitting, users may choose between frequentist and Bayesian paradigms. The frequentist approach yields point estimates of model parameters, while Bayesian inference provides full posterior distributions for comprehensive uncertainty quantification. Both paradigms support ordinary least-squares (OLS), weighted least-squares (WLS), and generalized least-squares (GLS, applied to FCS), corresponding to uniform, weighted, and correlated error structures, respectively. In Bayesian inference, integration over the parameter space yields the log-evidence (log *Z*), which serves as a quantitative criterion for model comparison. This metric naturally penalizes overly complex models by distributing probability mass over larger parameter regions, favoring simpler models unless the added complexity is justified by improved data fit.^60^

#### Visualization of Fitting Results

After fitting, the software overlays model curves onto the experimental ACF and can display residuals for both NLS and Bayesian approaches. For Bayesian inference, an additional uncertainty band is drawn around the median fit to represent the quantile ranges (QRs), which correspond to Bayesian credible intervals. Further visualization modes include posterior diagnostics, angular dependence plots in RICS, and spatially resolved parameter maps from sector analysis, enabling detection of heterogeneities in molecular transport.

### Application Examples

#### FCS—Single Trace

To demonstrate the FCS technique, a synthetic fluorescence trace was generated with a diffusion coefficient of *DC* = 100 μm^2^ s^–1^ (without triplet-state component), with the resulting intensity fluctuations shown in Figure 3A and the corresponding ACFs in Figure 3B. Both Bayesian inference and NLS fitting were applied to fit the ACF using a 3D isotropic diffusion model. Bayesian OLS (uniform errors) fitting estimated *DC* = 102.3 ± 1.5 μm^2^ s^–1^ (mean ± standard deviation, same notation is used throughout the *Examples*), in close agreement with *DC* value used in the simulations generating the synthetic data. The corresponding residuals are presented to illustrate the quality of the fit.

**Figure 3:**
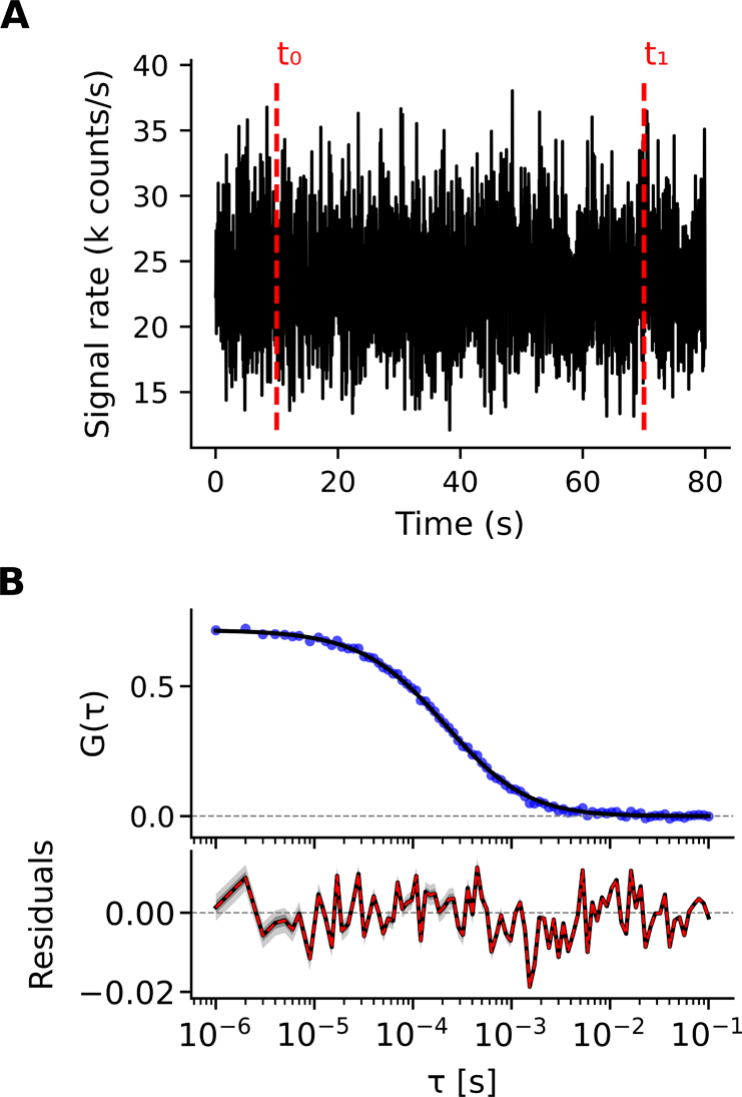
Demonstration of FCS using a synthetic fluorescence trace generated for particles with diffusion coefficient *DC* = 100 μm^2^s^–1^. (**A**) Time-resolved photon signal rate (in k counts/s), computed by temporal binning and normalization of photon counts, allowing visualization of fluorescence intensity fluctuations over time. The red dashed lines mark the selected time window (from *t*_0_ = 10 s to *t*_1_ = 70 s) used for autocorrelation analysis. (**B**) Top: Autocorrelation *G*(τ) (blue circles) plotted against lag time (τ), along with the median Bayesian ordinary least-squares (OLS) fit (black line) using a 3D isotropic diffusion model. Bottom: Posterior predictive residuals, with the black line representing the median and shaded regions, shown in dark and light gray, denote the 68.3% and 95.5% quantile ranges (QRs), respectively. The narrow uncertainty bands make the shading barely visible along the fit curve (top). The dashed red line denotes the residuals from the NLS method (with uniform errors) fit.

#### FCS—Multiple Traces

To illustrate the effect of errors and their correlation on fitting results, 2000 synthetic traces were generated for freely diffusing particles with diffusion coefficient *DC* = 20 μm^2^ s^–1^ (without triplet-state component). The covariance matrix of the resulting ACFs, shown in Supplementary Figure 1A (Supporting Information), reveals the correlation structure of noise across lag times. Both Bayesian inference and NLS fitting were applied using a 3D isotropic diffusion model. Supplementary Figure 1B shows the fit curve and residuals for the 3D model with Bayesian GLS fitting under correlated errors, yielding a diffusion coefficient of *DC* = 20.4 ± 1.8 μm^2^ s^–1^, in close agreement with *DC* value used in the simulations generating the synthetic data. Posterior distributions obtained from Bayesian GLS, WLS, and OLS approaches (Supplementary Figure 1D) highlight the impact of accounting for error correlation in parameter estimation. While OLS produces artificially narrow posteriors, GLS captures the variability in parameter estimates, yielding statistically rigorous inference. This capability to explicitly incorporate error correlations is an important feature of our framework and represents a significant improvement over commonly used OLS and WLS approaches.

In addition, four diffusion models were compared by Bayesian GLS fitting: 3D (log *Z* = 19.0 ± 0.3), 3D two-component (log *Z* = 8.2 ± 0.3), 3D with triplet-state (log *Z* = 16.8 ± 0.4), and 3D two-component with triplet-state (log *Z* = 6.3 ± 0.5). Bayesian model selection based on the log-evidence favored the simple 3D isotropic diffusion model, which provided the highest evidence score, demonstrating that the simplest model best explains the synthetic data.

To further evaluate performance under experimental conditions, Alexa Fluor 647-labeled Dextran 10K was measured diffusing in a 60% glycerol/water mixture. A total of 2000 traces were recorded, each with a trace duration of 2 min and a pixel time of 3 μs, and 1800 stable traces were selected for ACF calculation. Figure 4A shows the covariance matrix of the resulting ACFs. Both Bayesian inference and NLS fitting were applied using a 3D isotropic diffusion model with a triplet-state component which provided the highest evidence score and an experimentally measured PSF (Figure 4B). Bayesian GLS fitting with correlated errors estimated *DC* = 9.8 ± 1.1 μm^2^ s^–1^, *TSA* = 0.11 ± 0.02, and τ_*T*_ = 116 ± 78 μs. NLS fitting (with correlated errors) estimated similar parameters: *DC* = 10.5 ± 0.3 μm^2^ s^–1^, *TSA* = 0.11 ± 0.01, and τ_*T*_ = 40.9 ± 7.1 μs.

**Figure 4:**
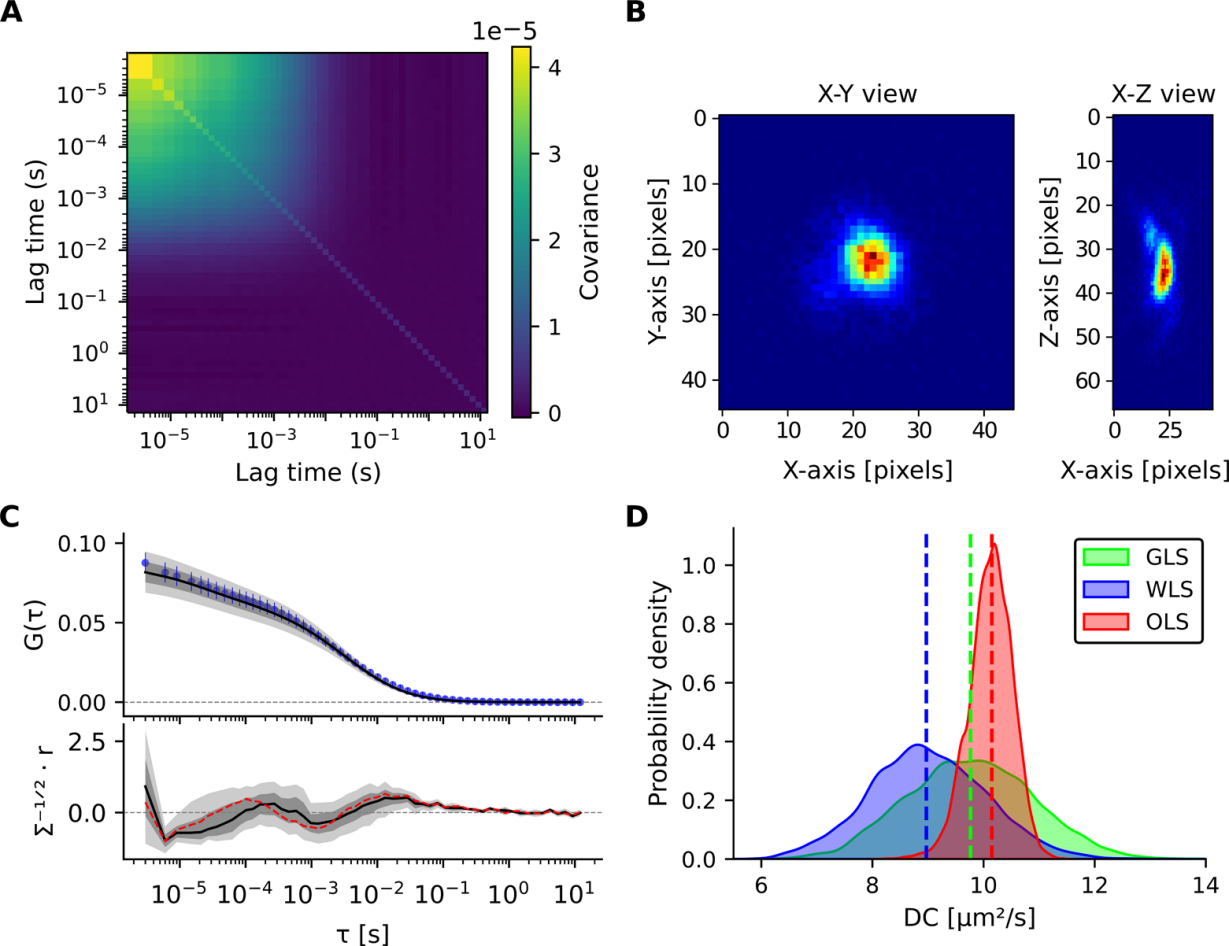
FCS measurements using 1800 experimental traces recorded for diffusion of Alexa-Dextran 10K in a 60% glycerol/water mixture. (**A**) Covariance matrix of the ACF computed from the 1800 individual traces, illustrating the correlation structure of noise across lag times. (**B**) Experimentally measured PSF used in model fitting, shown in *X*–*Y* and *X*–*Z* projections. Each pixel corresponds to a size of ≈50 nm. (**C**) Top: Mean autocorrelation *G*(τ) (blue circles) plotted versus τ, along with the median Bayesian GLS fit (black line) based on a 3D isotropic diffusion model including a triplet-state. Error bars represent the standard deviation (SD) of the mean. Bottom: Decorrelated residuals calculated from the ACF posterior predictive residual (*r*) and covariance matrix (Σ), with the black line showing the median residual. The dashed red line indicates the decorrelated residuals from the NLS method (with correlated errors) fit. Shaded regions (top and bottom), shown in dark and light gray, denote the 68.3% and 95.5% QRs, respectively. (**D**) Posterior distributions of *DC* obtained from three fitting approaches: Bayesian GLS (green, correlated errors), Bayesian weighted least-squares (WLS) (blue, weighted errors), and Bayesian OLS (red, uniform errors). Dashed lines indicate the medians of the respective distributions.

The estimated triplet-state relaxation time—or, in this case, the more general dark-state relaxation time—warrants discussion. Bayesian GLS fitting with correlated errors estimated τ_*T*_ = 116 ± 78 μs, while NLS fitting yielded τ_*T*_ = 40.9 ± 7.1 μs. The discrepancy between these estimates reflects the challenge of parameter estimation in systems with strong correlation structure. Examination of the posterior distribution (Figure S4A) reveals that the mode occurs near 40 μs, in good agreement with the NLS estimate as well as estimate of τ_*T*_ for Alexa Fluor 647 obtained in the same mixture by others.^75^ However, the distribution exhibits a long tail extending to higher values, which shifts the mean to larger values. In addition to the viscosity-dependent increase in τ_*T*_ observed previously^76,77^ this extended tail could be a reflection of complex interactions between the dye and glycerol.^78^

Posterior distributions obtained from Bayesian GLS, WLS, and OLS approaches (Figure 4D) again highlight that while OLS produces overly narrow posteriors, GLS appropriately accounts for correlated errors, yielding a more faithful representation of parameter uncertainty.

Four diffusion models were compared by Bayesian GLS fitting: 3D (log *Z* = 270.0 ± 0.2), 3D two-component (log *Z* = 278.0 ± 0.3), 3D with triplet-state (log *Z* = 279.4 ± 0.2), and 3D two-component with triplet-state (log *Z* = 277.1 ± 0.2). Bayesian model selection based on the log-evidence favored the 3D isotropic diffusion model with a triplet-state component, which yielded the highest evidence score among the tested models. The curve fits and corner plots of all four diffusion models are presented in Figures S2–S5 in the Supporting Information, while only the results corresponding to the model with the highest Bayesian evidence are shown in Figure 4.

The covariance matrix for Alexa-Dextran 10K in a 60% glycerol/water mixture, where diffusion is substantially slowed, exhibits pronounced off-diagonal terms, reflecting strong correlations between lag times (Figure 4A). Such correlations propagate into the posterior distributions of diffusion parameters, broadening the inferred uncertainty (Figure 4D). For comparison, measurements of Alexa-Dextran 10K in water, where diffusion is relatively faster, were performed under otherwise identical conditions. Similar to the 60% glycerol/water mixture, a total of 2000 traces were recorded, and 1800 stable traces were selected for ACF calculation. The corresponding covariance matrix displays much weaker off-diagonal correlations (Figure S6A). Both Bayesian inference and NLS fitting were applied using a 3D isotropic diffusion model with a triplet-state component and an experimentally measured PSF (Figure 4B). Bayesian GLS fitting with correlated errors estimated *DC* = 84.8 ± 3.1 μm^2^ s^–1^, *TSA* = 0.19 ± 0.01, and τ_*T*_ = 10.2 ± 2.5 μs. NLS fitting (with correlated errors) estimated similar parameters: *DC* = 85.1 ± 1.5 μm^2^ s^–1^, *TSA* = 0.19 ± 0.01, and τ_*T*_ = 9.4 ± 0.9 μs. In contrast to the long τ_*T*_ observed in the glycerol/water mixture, both Bayesian GLS and NLS fitting for Alexa-Dextran 10K in water yielded consistent and smaller triplet-state relaxation times, accompanied by narrower posterior distributions and lower parameter uncertainty (Figure S6C).

Posterior distributions obtained from Bayesian GLS, WLS, and OLS approaches (Figure S6D) exhibit more similar shapes compared to the case with strong off-diagonal terms, reflecting the reduced influence of noise correlations. These results highlight the direct relationship between diffusion dynamics, the structure of noise correlations, and the reliability of the estimated diffusion coefficients. The corresponding corner plot and fitted ACF curves are shown in Figure S6.

#### RICS—Laser Scanning Speeds

The software supports analysis of RICS data acquired at different laser scanning speeds. To illustrate this, diffusion of Alexa-Dextran 10K in water was recorded (600 RICS frames) within a 20 × 20 μm^2^ scanning area at three scanning speeds, fast, medium, and slow, corresponding to 1000, 1732, and 3000 pixels per line along the *X*-axis (line times of 2, 3.5, and 6 ms, including flyback), respectively. Slower scanning enables acquisition of more pixels per line, providing finer spatial sampling (Figure 5). Both Bayesian inference and NLS fitting were applied to fit the ACF from three experimental conditions using a 3D isotropic diffusion model that included a triplet-state component. Bayesian WLS fitting with weighted errors yielded *DC* = 81 ± 17 μm^2^ s^–1^, *TSA* = 0.29 ± 0.13, and τ_*T*_ = 3.41 ± 2.05 μs. NLS fitting (with weighted errors) estimated similar parameters: *DC* = 73.9 ± 1.3 μm^2^ s^–1^, *TSA* = 0.34 ± 0.01, and τ_*T*_ = 1.94 ± 0.14 μs. The experimentally measured PSF shown in Figure 4B was used for all fits. Posterior distributions of *DC*, obtained using Bayesian WLS and OLS approaches (Figure 5H) highlight the impact of accounting for measurement errors in parameter estimation.

**Figure 5:**
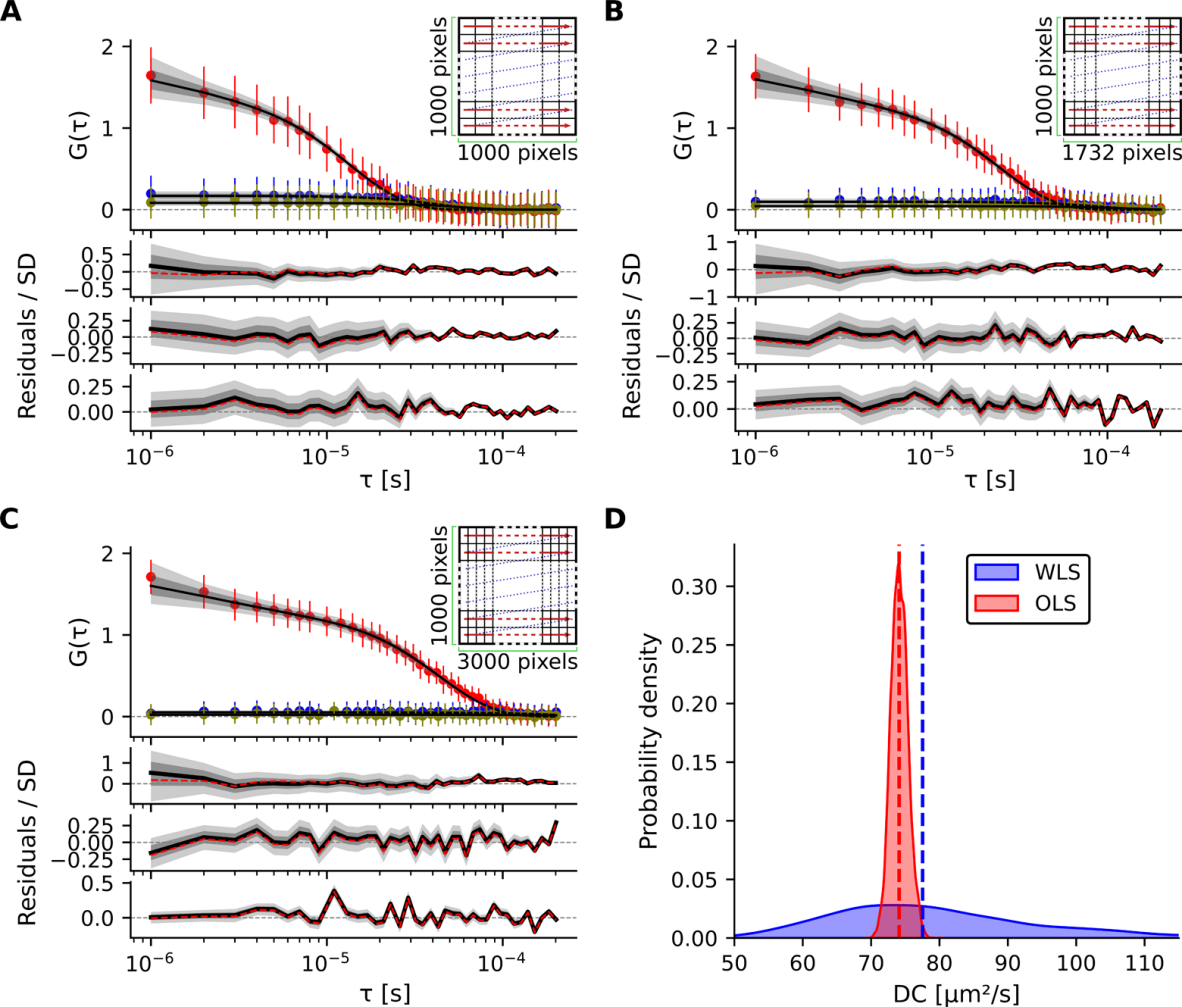
RICS analysis at varying laser scanning speeds, recorded for diffusion of Alexa-Dextran 10K in water. (**A**, **B**, **C**) Schematic illustrations of fast, medium, and slow scanning speeds, corresponding to line times of 2, 3.5, and 6 ms (including flyback), respectively, and corresponding analysis results for each scanning scheme. Red arrows indicate the laser scanning direction. The mean autocorrelation *G*(τ) (colored circles) of three lines plotted versus τ, along with the median Bayesian WLS fit (black line) based on a 3D isotropic diffusion model including a triplet-state component. Error bars represent the SD of the mean. Standardized posterior predictive residuals, with the black line showing the median residual are shown in bottom. The dashed red lines indicate the standardized residuals from the NLS method (weighted errors) fit. Shaded regions, shown in dark and light gray, denote the 68.3% and 95.5% QRs, respectively. (**D**) Posterior distributions of *DC* obtained from two fitting approaches: Bayesian WLS (blue, weighted errors) and Bayesian OLS (red, uniform errors). Dashed lines indicate the medians of the respective distributions.

#### RICS—Laser Scanning Angles

The software enables analysis of RICS data acquired at multiple laser scanning angles, which is essential for characterizing anisotropic diffusion in complex cellular environments. As a demonstration, diffusion of Alexa-Dextran 10K in a single live rat cardiomyocyte (CM) was recorded within a 20 × 20 μm^2^ scanning area (Figure 6A) at seven different scanning angles from 0° to 180° in 30° steps (Figure 6B) and at three different scanning speeds (21 conditions in total). A total of 4560 RICS frames were recorded; the first 568 frames were excluded due to unstable intensity. Both Bayesian inference and NLS fitting approaches were applied using a 3D anisotropic diffusion model that included a triplet-state component, accounting for diffusion along the *X*-axis (longitudinal) and *Y*-axis (transverse), with diffusion along the *Z*-axis assumed equal to the transverse component. Bayesian WLS fitting with weighted errors yielded *DC*_*x*_ = 19.2 ± 5.7 μm^2^ s^–1^, *DC*_*y*_ = 13.2 ± 3.3 μm^2^ s^–1^, *TSA* = 0.34 ± 0.05, and τ_*T*_ = 4.5 ± 5.9 μs. NLS fitting (with weighted errors) estimated similar parameters: *DC*_*x*_ = 18.5 ± 0.6 μm^2^ s^–1^, *DC*_*y*_ = 13.4 ± 0.4 μm^2^ s^–1^, *TSA* = 0.35 ± 0.01, and τ_*T*_ = 2.8 ± 0.1 μs. The experimentally measured PSF shown in Figure 4B was used for all fits. The obtained diffusion coefficients for CMs are similar to our earlier estimates (*DC*_*x*_ = 19 ± 3 μm^2^ s^–1^ and *DC*_*y*_ = 16 ± 2 μm^2^ s^–1^).^27^ Representative curve fits and residuals for two example conditions −60° and 90° angles at medium scanning speed—are shown in Figure 6C and D. Posterior distributions of *DC*_*x*_ and *DC*_*y*_ from all 21 experimental conditions, obtained using Bayesian WLS and OLS approaches (Figure 6E, F), highlight the importance of explicitly accounting for measurement errors in anisotropic diffusion parameter estimation. These results demonstrate that the software enables rigorous Bayesian inference for anisotropic diffusion analysis in living cells.

**Figure 6:**
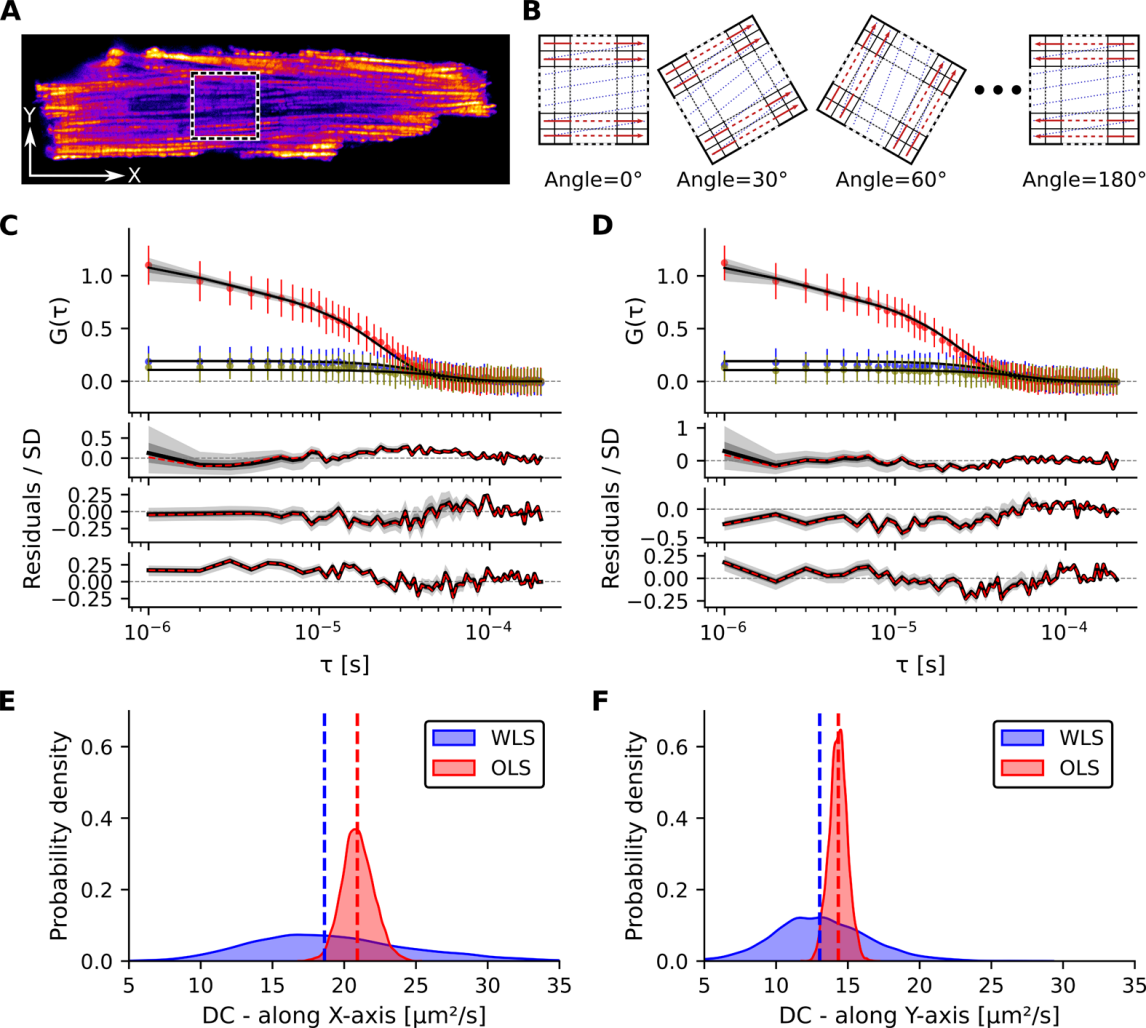
Anisotropic diffusion analysis using RICS with multiple scanning angles and speeds, recorded for diffusion of Alexa-Dextran 10K in a single rat cardiomyocyte (CM). (**A**) Confocal image of a live rat CM mechanically permeabilized by localized poking, shown in pseudocolor depicting mitochondria (Mitotracker Green). White arrows indicate the physical *X* and *Y* directions. The dashed square marks the 20 × 20 μm^2^ scanning area. (**B**) Schematic illustrations of different scanning angles from 0° to 180° in 30° steps. (**C**, **D**) Top: Mean autocorrelation *G*(τ) (colored circles) of three lines plotted versus τ, along with the median Bayesian WLS fits (black lines) based on a 3D anisotropic diffusion model including a triplet-state component, for the 60° and 90° scanning angles, respectively, at medium scanning speed (line time of 3.5 ms). Error bars represent the SD of the mean. Bottom: Standardized posterior predictive residuals, with the black line showing the median residual. The dashed red lines indicate standardized residuals from the NLS method (weighted errors) fit. Shaded regions, shown in dark and light gray, denote the 68.3% and 95.5% QRs, respectively. (**E**, **F**) Posterior distributions of *DC_x_* and *DC_y_* from all 21 experimental conditions, obtained from two fitting approaches: Bayesian WLS (blue) and Bayesian OLS (red). Dashed lines indicate the medians of the respective distributions.

#### Spatial Mapping of RICS-Derived Parameters

The software enables spatial mapping of molecular transport by dividing RICS images into user-defined sectors and computing the ACF within each region. This approach provides flexibility in resolution and supports detailed characterization of parameter heterogeneity across cellular areas of interest. As an example of the second configuration (larger-area self-correlation in Figure 1B), Figure 7 illustrates the spatial mapping of Alexa-Dextran 10K in a fixed mouse CM. The cell was mechanically permeabilized by localized poking and stained with Mitotracker Green to visualize mitochondria (Figure 7A). The pseudocolor image highlights the cellular structure, with a dashed square indicating the 20 × 20 μm^2^ scanning area selected for RICS analysis. As shown, the scanning window encompasses both part of the cell and the surrounding solution, enabling direct comparison of intracellular and extracellular diffusion within the same acquisition. A total of 3700 RICS frames were recorded; the first 700 frames were excluded due to unstable intensity. Subsequent fitting was performed using a 3D isotropic diffusion model including a triplet-state component, with the PSF experimentally determined in Figure 4B. For this analysis, three scanning speeds (2, 3.5, and 6 ms) were applied at a single scanning angle of 0°. The resulting diffusion coefficient map (Figure 7B) and concentration map (Figure 7C) clearly illustrate local variations in molecular mobility and Alexa-Dextran 10K concentration within the scanned area, which spans both intracellular and extracellular solution regions. This example demonstrates the ability of the software to transform RICS recordings into quantitative parameter maps, providing both a spatially resolved view of molecular transport and insight into the heterogeneity of the intracellular environment.

**Figure 7:**
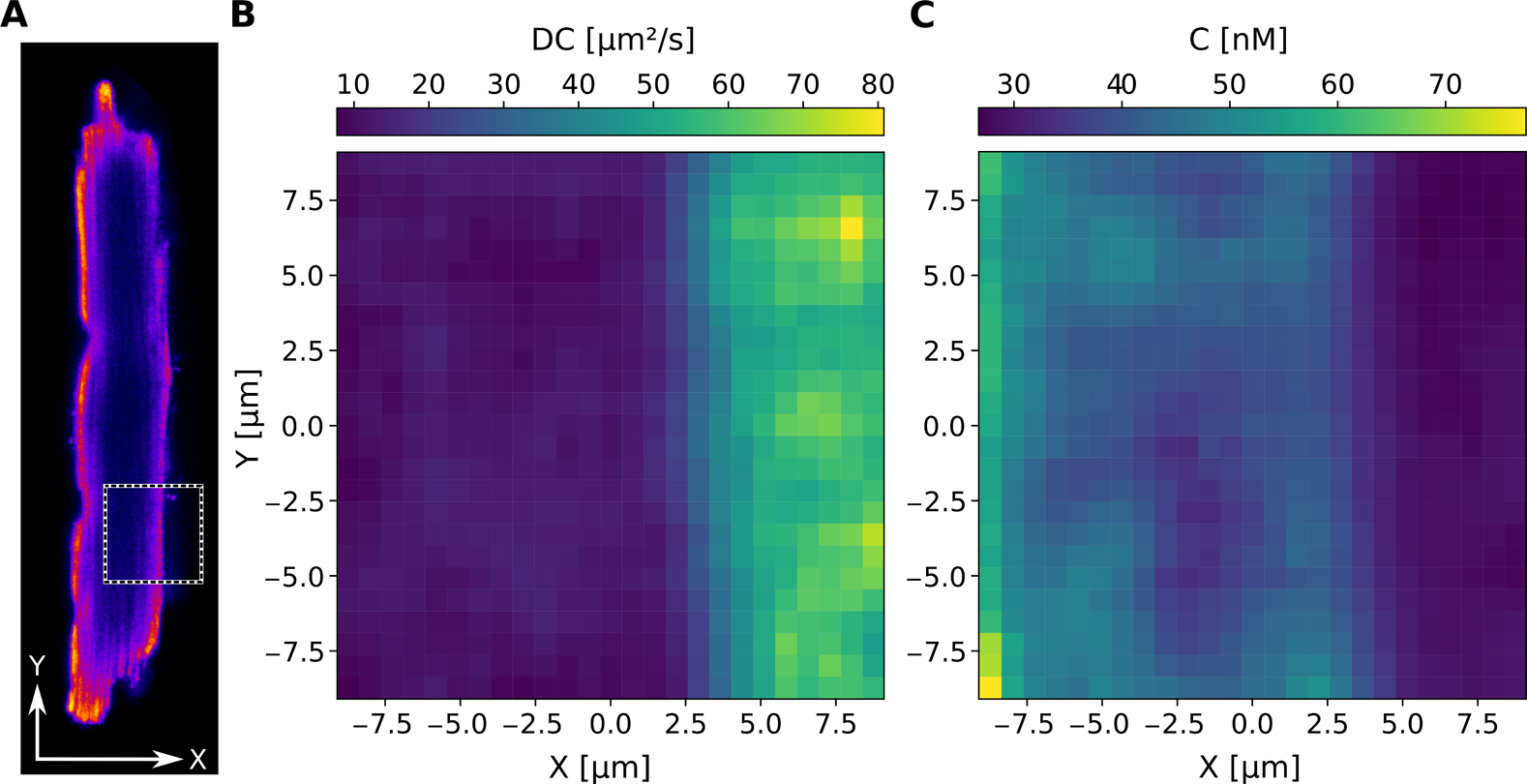
Spatial mapping of RICS-derived parameters, recorded for diffusion of Alexa-Dextran 10K in a single mouse CM at C ≈ 50 nM (Alexa-Dextran 10K + solution + cell). (**A**) The confocal image of a fixed mouse CM, shown in pseudocolor depicting mitochondria (Mitotracker Green), with white arrows indicating the physical *X* and *Y* directions. The dashed square indicates the 20 × 20 μm^2^ scanning area used for RICS analysis, covering both a portion of the cell and the surrounding solution. (**B**) Diffusion coefficient map of the recorded scanning area, and (**C**) corresponding concentration map. Each map consists of 25 × 25 equally sized spatial sectors (0.8 × 0.8 μm^2^). For each sector, ACF was computed using the fluorescence signals within a 3 × 3 μm^2^ area (larger-area self-correlation), and fitted using a 3D isotropic diffusion model including a triplet-state component to extract the respective parameter values. For fitting, the NLS method (uniform errors) was applied, using the PSF determined in Figure 4B. Color bars indicate the parameter scales in physical units.

### Performance Benchmarks

ACF calculation performance was evaluated using the hardware and software specifications described in *Methods*. GPU acceleration substantially improves ACF calculation speed (Table 1), achieving 19-fold speedup for FCS analysis of 100 traces (100 s each with 10^8^ time points per trace at 1 μs resolution) and 8-fold speedup for RICS processing of 350 images (1000 × 1732 pixels with 1 μs pixel dwell time). The greater FCS acceleration reflects larger data set size (10^10^ total time points vs 6 × 10^8^ pixels). These speedups are critical for high-throughput experiments, reducing processing from hours to minutes.

**Table 1:** GPU Vs CPU Performance for ACF Calculation

Data set	CPU (s)	GPU (s)	Speedup
FCS (100 traces, 10^8^ points each)	799.1	41.8	19×
RICS (350 images, 1000 × 1732 pixels)	38.6	4.8	8×

The impact of using analytical Gaussian or experimentally measured PSF was evaluated using the same hardware and software. Experimentally measured PSFs substantially improve fit quality compared to the use of analytical Gaussian PSF (Table 2). For FCS analysis of 200 traces from Figure 4 (using NLS-GLS fitting with correlated errors) and RICS analysis of 600 frames from Figure 5 (using NLS-WLS fitting with weighted errors), measured PSFs led to a 2–6-fold decrease in the chi-squared statistic (χ^2^(θ̂)) relative to Gaussian approximations, demonstrating the importance of capturing real optical aberrations and asymmetries. The Gaussian PSF parameters (ω_*x*_, ω_*y*_, ω_*z*_) were obtained by fitting the same experimentally measured 3D PSF to a Gaussian function, ensuring a fair comparison between analytical Gaussian and experimentally measured PSF. The computational overhead scales with data set complexity: for Figure 5 with 3 scan configurations, use of the full experimental PSF during fitting required 3.8 s versus 1.3 s for models that use Gaussian approximation for PSF; for the larger data set in Figure 6 with 21 configurations (3 speeds × 7 angles), this increased to 30 s versus 1.4 s. The FCS benchmark used the first 200 traces from Figure 4, as larger data sets would introduce substantial preprocessing overhead (covariance matrix computation and memory management) that could obscure PSF-specific performance differences.

**Table 2:** Comparison of Model Fits Using Analytical Gaussian and Experimentally Measured PSF: Fit Quality and Computational Cost

Data set	PSF	PSF Size	χ^2^(θ̂)^a^	Time (s)
FCS (200 traces)	Full measured	(*x*, *y*, *z*): 35 × 35 × 43 voxels	10.32	3.5
	Downsampled 2×	(*x*, *y*, *z*): 17 × 17 × 21 voxels	20.27	3.1
	Downsampled 3×	(*x*, *y*, *z*): 11 × 11 × 14 voxels	23.52	2.8
	Gaussian	(ω_*x*_, ω_*y*_, ω_*z*_): (0.36, 0.38, 1.08) μm	57.46	2.4
RICS (600 frames)	Full measured	(*x*, *y*, *z*): 35 × 35 × 43 voxels	3.04	3.8
	Downsampled 2×	(*x*, *y*, *z*): 17 × 17 × 21 voxels	3.23	3.4
	Downsampled 3×	(*x*, *y*, *z*): 11 × 11 × 14 voxels	4.13	3.4
	Gaussian	(ω_*x*_, ω_*y*_, ω_*z*_): (0.36, 0.38, 1.08) μm	6.55	1.3

a The chi-squared statistic (χ^2^(θ̂)) computed using decorrelated residuals (GLS fitting) for FCS and standardized residuals (WLS fitting) for RICS.

To understand the computational overhead of using experimentally measured PSF for fitting ACF, we quantified its contribution to the fitting stage of the analysis workflow, which consists of data loading, model fitting, and output writing. Data loading and output writing introduce a fixed overhead that is independent of the PSF used. For fits using Gaussian PSFs, the model fitting step represents only a negligible fraction of total fitting runtime (<2%). In contrast, when using experimentally measured PSFs, model fitting becomes a substantial component of the fitting stage for complex data sets (e.g., ∼60% of fitting time for RICS with 3 configurations and ∼95% for 21 configurations), while remaining more moderate for FCS (e.g., ∼20% for FCS data set in Table 2). Overall, using experimentally measured PSFs during fitting substantially increases the fitting-stage duration.

To balance accuracy and computational efficiency, the software allows flexible voxel downsampling of the PSF, reducing resolution in the lateral (*x*, *y*) or axial (*z*) dimensions independently while preserving the PSF’s overall shape and lowering computational cost. Table 2 demonstrates uniform downsampling by factors of 2× and 3× in all three dimensions, showing that moderate downsampling reduces numerical integration cost while preserving essential optical characteristics. For high-resolution PSFs with hundreds of voxels per dimension, downsampling provides more pronounced computational benefits. This flexibility allows users to optimize the accuracy-speed trade-off based on PSF resolution, data set characteristics, and computational resources.

An important consideration when selecting a PSF representation for fitting is how analysis time contributes to the overall experimental workflow. The computational analysis phase, including GPU-accelerated ACF calculation and model fitting, is substantially shorter than the data acquisition time. For example, irrespective of whether analytical or experimental PSF is used, analyzing a single 120 s FCS trace requires only 2–3 s of computation (ACF calculation plus fitting), while large data sets such as 200 FCS traces (6.5 h of acquisition) or 600 RICS images (1 h of acquisition) require on the order of 10 s to analyze, corresponding to well below 1% of the acquisition time. Even for the most computationally demanding data set (21 RICS configurations; ∼10 h of acquisition), the total analysis time is on the order of 1 min. Thus, although fitting with experimentally measured PSFs increases the computational cost relative to models using Gaussian PSFs, the absolute runtime remains acceptable for routine analysis, particularly given the substantial improvements in fit quality demonstrated in Table 2.

## Discussion

We present a unified open-source Python platform for the analysis of FCS and RICS experiments to extract molecular transport parameters including diffusion coefficients, triplet dynamics, and concentrations from fluorescence fluctuation data. The platform integrates GPU-accelerated ACF calculation, rigorous statistical inference (frequentist and Bayesian approaches), support for experimentally measured PSFs, and analysis of anisotropic diffusion in cellular environments, addressing key limitations in current correlation spectroscopy workflows.

The analysis of FCS and RICS data is inherently challenging due to biological heterogeneity, experimental noise, and the need for statistically robust parameter extraction. The developed platform systematically addresses these challenges through several key features, as discussed below.

### Advanced Features for Complex Biological Systems

The ability to incorporate experimentally measured PSFs is particularly important in systems where optical aberrations significantly affect parameter extraction. The software supports both Gaussian and experimentally measured PSFs. While Gaussian PSFs are widely used for simplicity, experimentally measured PSFs yield more realistic fits by capturing aberrations, asymmetries, and other optical characteristics that analytical models cannot represent, as demonstrated in Table 2. However, experimentally measured PSFs demand greater computational effort due to their 3D complexity and higher spatial sampling requirements. To balance accuracy and efficiency, the software allows voxel downsampling of the PSF, reducing computational cost while preserving overall PSF shape.

Multiangle RICS analysis enables characterization of anisotropic diffusion in structured biological samples such as cardiomyocytes, where molecular transport is spatially constrained. The platform enables analysis of experiments where multiple scanning protocols (different angles, speeds) were performed on the same sample, leveraging complementary information to reduce parameter degeneracy. When ACFs are acquired at different scanning speeds, the platform validates model consistency by jointly fitting across all speeds. When ACFs are acquired at different scanning angles, the platform estimates anisotropic diffusion within cells. As shown in Figure 6, the angularly resolved RICS can uncover directional dependencies of diffusion processes that remain hidden in isotropic analyses.^27,37^

Furthermore, splitting RICS images into smaller regions (Figure 1) enables spatial mapping of diffusion and concentration (Figure 7). Such maps reveal subcellular domains with slower diffusion or higher concentration, which often correlate with structural features such as organelles or cytoskeletal barriers.

### Model Selection Strategies and Challenges

Fitting ACF data requires selecting among three model categories: statistical framework (Bayesian inference or NLS), error structure (OLS/WLS/GLS), and diffusion type (isotropic/anisotropic, single/two-component, ±triplet kinetics). These choices strongly influence parameter estimation and uncertainty quantification.

Bayesian inference and NLS often yield similar mean parameter estimates but differ substantially in uncertainty quantification, as demonstrated in our experimental results where mean values are comparable while Bayesian standard deviations are substantially broader, more accurately reflecting parameter uncertainty. Bayesian inference yields full posterior distributions that reveal parameter correlations via corner plots and enable principled model comparison through evidence evaluation. The posterior distributions naturally quantify uncertainty through their spread, with broader distributions indicating greater parameter uncertainty and narrower distributions indicating well-constrained parameters. However, Bayesian inference requires substantially longer computation than NLS—typically 2 to several hundred times longer depending on model complexity and data set size—due to the need for extensive posterior sampling via nested sampling algorithms.

The choice between statistical frameworks depends on research priorities. NLS is appropriate for rapid parameter screening, high-throughput analysis, or when point estimates with approximate uncertainties suffice. Bayesian inference is essential when rigorous uncertainty quantification is required, when comparing competing models, when parameter correlations must be characterized, or when decisions depend critically on confidence in parameter estimates. For systematic studies requiring model selection across many data sets, the computational investment in Bayesian inference is typically justified by the reliability of the resulting conclusions.

For distinguishing among diffusion models (single vs two-component, ±triplet state), two quantitative approaches are available: Bayesian model comparison via log evidence (log *Z*) and frequentist hypothesis testing using the F-test. Bayesian evidence provides the most robust criterion, particularly when using GLS with the full covariance matrix. Following standard interpretation scales for natural logarithm differences^59,79^ |Δlog *Z*| < 1 indicates models are effectively indistinguishable, |Δlog *Z*| = 1–3 provides positive evidence, |Δlog *Z*| = 3–5 constitutes strong evidence, and |Δlog *Z*| > 5 represents very strong evidence favoring one model over another. The log *Z* values reported by the software include uncertainty estimates from the nested sampling procedure, allowing rigorous error propagation in model comparison.^60^

Nested diffusion models can be compared using the F-test, which assesses whether the reduction in residual variance achieved by a more complex model is statistically significant. The software reports the reduced chi-squared for each fitted model, from which the F-statistic can be directly constructed. Unlike Bayesian evidence, the F-test is restricted to nested models and assumes Gaussian residuals.^80^

The Bayesian evidence is most effective with GLS using decorrelated residuals; WLS with standardized residuals provides intermediate reliability, while OLS can lead to incorrect model selection due to neglected error correlations. GLS accounts for both heteroscedasticity and temporal correlations, providing the most reliable metrics when covariance can be estimated from replicate measurements. WLS accounts for heteroscedastic errors using point-wise variance estimates, offering a practical compromise applicable to both FCS and RICS. OLS assumes identical error across all ACF points and independence between measurements, leading to artificially narrow parameter distributions and an underestimation of uncertainty, but remains useful for rapid preliminary analysis or when only single measurements exist. For RICS data, GLS is currently not applicable (see *Limitations and Future Directions* below), restricting model comparison to WLS-based metrics or visual residual assessment.

The importance of proper error modeling is illustrated with synthetic data (Figure S1), where traces of freely diffusing particles without triplet kinetics were generated. When multiple traces were fitted using GLS with the full covariance matrix, the correct 3D isotropic model yielded the highest Bayesian evidence. However, fitting a single trace with OLS did not allow reliable model discrimination: the 3D model with triplet state (log *Z* = −24.7 ± 0.4) and the true 3D isotropic model (log *Z* = −25.6 ± 0.4) yielded statistically indistinguishable evidence (|Δlog *Z*| = 0.9). This demonstrates how ignoring noise structure can lead to incorrect model selection, consistent with previous reports emphasizing the importance of proper error modeling in fluctuation spectroscopy.^32,40^

Determining whether diffusion is isotropic or anisotropic requires multiangle RICS measurements. In isotropic systems (e.g., solution-phase experiments), anisotropic fitting should yield nearly identical diffusion coefficients across all scan directions (*DC*_*x*_ ≈ *DC*_*y*_ within uncertainty). Anisotropic systems such as cardiomyocytes show clear directional dependence (Figure 6). However, weak anisotropy can be challenging to establish confidently, as directional differences may approach the magnitude of systematic uncertainties in PSF characterization or be obscured by noise correlations. A key limitation is that PSFs are characterized in solution rather than within live cells, as described in PSF Characterization. This measured PSF is then applied to fit both solution and live-cell experiments. Refractive index mismatches and optical aberrations introduced by cellular structures can alter the PSF shape compared to solution measurements, potentially contributing additional systematic uncertainty to extracted diffusion coefficients. Distinguishing genuine anisotropy from experimental artifacts—including PSF-related systematic errors—requires careful experimental design with multiple scan angles and sufficient statistical power.

### Comparison with Existing Software

Several open-source tools have been developed for FCS and RICS analysis over the past two decades, each contributing valuable capabilities while exhibiting specific limitations (Table 3). ImFCS introduced imaging-based correlation methods supporting both point FCS and scanning FCS configurations.^42^ PyCorrFit provided a Python-based framework for generic FCS data evaluation with multiple diffusion models and WLS fitting.^45^ FCSlib offers FCS and scanning FCS analysis in R with functions for mobility and molecular brightness estimation.^48^ Specialized Python tools for optimized FCS and scanning FCS analysis have been developed for conventional confocal microscopy data.^46^ The Imaging FCS plugin for Fiji/ImageJ implements GPU-accelerated with PSF calibration capabilities, supporting FCS and scanning FCS.^50^ For RICS analysis, several implementations exist within the Fiji/ImageJ ecosystem^43,44^ providing accessible tools for spatial correlation analysis. Several MATLAB-based tools have been developed for fluorescence fluctuation analysis. These include the PAM framework, which integrates FCS and RICS analysis with ensemble and single-molecule fluorescence methods.^81^ Another example is a quantitative 4D imaging approach that uses FCS calibration.^47^ Additionally, fcsSOFI provides the spatiotemporal correlation method for scanning FCS, featuring GPU acceleration for the fitting stage.^49^ While these MATLAB-based tools are open-source, their dependence on MATLAB as a proprietary environment limits broad accessibility.

**Table 3:**
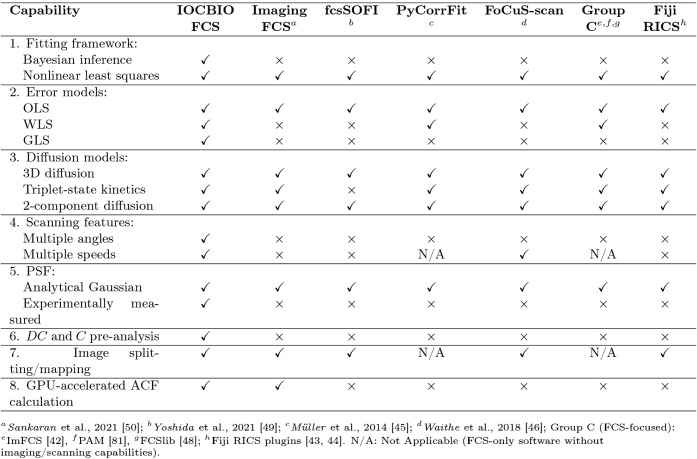
Comparison of Open-Source FCS/RICS Analysis Platforms

Table 3 systematically compares correlation-based FCS and RICS platforms across key analytical capabilities. While existing software provide diverse features, critical limitations remain. None of these tools support multiple-angle RICS analysis for characterizing anisotropic diffusion in biological systems. While PSF calibration has been implemented in some packages^50^ based on our knowledge no open-source tool incorporates experimentally measured 3D PSFs into the fitting procedure, and available frameworks remain limited to analytical Gaussian approximations. Only Imaging FCS currently supports GPU acceleration for ACF calculation, enabling order-of-magnitude speedups.^50^ Based on our knowledge, Bayesian inference for fitting ACF data is not available in any of the existing packages, and our implementation is unique in this regard. Furthermore, while available software relies on OLS or WLS for NLS fitting, our framework extends both Bayesian and NLS fitting by providing support for OLS, WLS, and GLS. Additionally, the platform introduces diffusion-concentration pre-analysis and filtering to identify and exclude poor-quality measurements before main parameter estimation. Our platform unifies FCS and RICS analysis within a single framework, with the flexibility to perform scanning FCS by setting the line scan displacement to zero (Δ*y* = 0) in the RICS, thereby covering the full spectrum of correlation-based fluorescence techniques.

### Machine Learning and Direct Trace Analysis

In recent years, machine learning (ML) and deep learning (DL) techniques have been explored for FCS and imaging-FCS applications.^82^ *Tang* et al. introduced FCSNet and ImFCSNet, convolutional neural networks (CNN) trained on simulated data that predict diffusion parameters directly from intensity traces, reducing data requirements and enabling near real-time inference.^83^ ML approaches have also been applied for artifact filtering^36^ and anomalous diffusion classification.^84^ However, these approaches face limitations: training on simulated data may not generalize to real experimental conditions that vary from one microscope to another; learned representations lack physical interpretability, hindering failure diagnosis; and integrated uncertainty quantification is limited. ML/DL methods are thus complementary to correlation-based approaches—excelling at rapid screening when training data exist, while correlation methods provide interpretable, physically grounded parameters with rigorous statistical inference.

An emerging alternative to conventional FCS is the class of methods based on direct fitting of experimental data using stochastic models. One of these methods, fluorescence intensity trace statistical analysis (FITSA), is a Bayesian framework that directly analyzes fluorescence intensity traces rather than derived autocorrelations.^65^ Earlier Bayesian approaches introduced by *Jazani* and colleagues^85–88^ established the conceptual basis for direct statistical analysis of photon traces, but their initial implementations^86^ faced challenges related to computational efficiency and convergence.^65^ FITSA addresses these limitations by offering faster and more stable convergence, enabling robust parameter estimation from substantially shorter measurements than FCS, minimizing laser exposure and phototoxicity, and accounting for statistical dependencies often overlooked in standard FCS fitting. By requiring fewer photons and avoiding problematic assumptions of autocorrelation analysis, FITSA and similar methods represent a major conceptual advance in fluctuation spectroscopy. However, FITSA is currently limited to relatively simple diffusion models (e.g., 3D single-component isotropic diffusion without triplet dynamics), requires low fluorophore concentrations, and is computationally demanding for more complex data sets. Addressing these FITSA issues will take some time, and this class of methods still has to prove itself in the field to fully fulfill their great potential.

The choice among approaches depends on experimental constraints and scientific goals. Our correlation-based platform is optimal for anisotropic diffusion characterization, multicomponent diffusion, triplet-state kinetics, spatial parameter mapping, and moderate-to-high concentrations. Current FITSA implementation excels for brief measurements at low concentrations with simple single-component isotropic diffusion. ML/DL methods suit real-time analysis and classification tasks. As direct trace analysis extend to handle anisotropic diffusion, multicomponent models, and higher concentrations, they may provide future alternatives to correlation-based analysis for broader applications.

### Limitations and Future Directions

Despite the advances described above, several limitations remain, ranging from practical software constraints to fundamental experimental challenges inherent to FCS and RICS. The software currently relies on HDF5 format for standardized input and output of raw data, correlation results, and fitted parameters. While HDF5 is an open format, future development is needed to facilitate conversion of user data sets into this format. This development is planned to be demand-driven, with either direct data import support or dedicated converters created through collaboration between developers and users.

Bayesian inference, while powerful for model selection and quantifying parameter uncertainty, remains computationally demanding for complex models. Future development could extend GPU acceleration to the Bayesian inference framework to improve computational efficiency.^89^

To take full advantage of GLS-based fitting, the covariance matrix must be estimated. This enables full implementation of Bayesian GLS fitting with rigorous uncertainty quantification and allows model comparison while accounting for correlated errors. In our current implementation, GLS-based fitting is supported only for FCS, where a single 1D intensity trace can be repeated thousands of times (much larger than the dimension of the ACF) to robustly estimate the covariance across lag times. Such estimation imposes several practical challenges to ensure the stability of the experimental setup. In the example shown in Figure 4, experiments took approximately 100 h. Over this time window, special precautions were necessary to prevent temperature fluctuations due to air conditioning and to minimize airflow variability in the microscopy room caused by ventilation cycles. These experiments were performed in solution and would be impossible—or at least exceptionally difficult—to perform on live cells due to phototoxicity, cell movement, and viability constraints. When such large numbers of repeats are not available, shrinkage-based approaches for estimating noise correlations from multiple ACFs can be applied.^40^ However, whether this shrinkage fully reflects covariances through its approximation of the full covariance matrix is unclear. An alternative is to implement segmented and randomized ACF approaches, which reduce correlations between data points and allow standard goodness-of-fit evaluation with shorter experiments.^32^ While not currently implemented, this is a promising approach for future development.

GLS fitting is not implemented for RICS due to experimental limitations, which are even more pronounced than for FCS. For RICS, after recording image stacks (e.g., 2000 frames of 1000 × 1000 pixels), the 2D spatiotemporal ACF is cropped, and one quadrant containing positive time lags is selected for fitting. By default, this yields three correlation lines with approximately 100 ACF points each, which are flattened into a 1D vector (about 300 points total). Reliable estimation of such a covariance matrix would require thousands of independent RICS frames acquired under identical conditions (frame size, scanning speed, and angle), which we expect is practically impossible in live-cell experiments. Additionally, RICS data acquisition is slower than FCS due to flyback periods during scanning when no data are collected, further extending the required experimental time.

As discussed earlier, PSFs are currently characterized in solution rather than within live cells. Future development of methods for in-cell PSF characterization would improve accuracy, particularly for detecting anisotropy where systematic PSF errors can approach or exceed the magnitude of directional diffusion differences. As a current practical solution, the platform allows PSF scale as an additional free parameter during fitting to accommodate calibration uncertainties or systematic PSF size deviations without requiring remeasurement.

## Conclusion

The presented open-source Python platform (IOCBIO FCS) unifies the analysis of FCS and RICS within a statistically rigorous and computationally efficient framework. By integrating GPU-accelerated ACF computation, support for arbitrary scan angles and speeds, and image splitting for spatial parameter mapping, the software enables analysis of large data sets and provides the ability to study anisotropic diffusion. Incorporation of experimentally measured PSFs, advanced filtering strategies, and robust fitting approaches—including both frequentist and Bayesian inference under explicit noise models—distinguishes the platform from existing tools. Comprehensive visualization of fitted results, residuals, posterior distributions, and spatial maps further ensures reproducibility and interpretability. Together, these capabilities establish a versatile framework for quantitative correlation-based fluorescence methods, with broad relevance to biophysics, biochemistry, and cellular biology research.

## Statistics and Reproducibility

All FCS and RICS data were analyzed using the statistical frameworks implemented in the IOCBIO FCS software. Parameter estimation was performed using NLS and Bayesian inference, with uncertainty quantified through posterior distributions. Model comparison was based on the Bayesian evidence (*Z*). The number of traces or image frames used for analysis is indicated in the *Results* section.

## Supplementary Material



## Data Availability

The data supporting the findings of this study are available from the corresponding author upon reasonable request. Representative FCS and RICS example data sets are accessible at: https://iocbio.gitlab.io/fcs/example-datasets. The source code and documentation are publicly available through the GitLab project at: https://iocbio.gitlab.io/fcs/.

## Notes

Additional synthetic FCS validation data and covariance structure of autocorrelation noise; Bayesian model comparison of one- and two-component diffusion models with and without triplet-state contributions; additional experimental FCS analysis of Alexa Fluor 647-labeled Dextran; corresponding corner plots for all analyses (Figures S1–S6) (PDF)
