# Supplementary material for: A Unified Platform for FCS and RICS Analysis with Advanced Statistical Inference
Source: ACS Omega. 2026 Mar 16;11(12):19201–19. doi: 10.1021/acsomega.5c12269 (PMC13044634; doi:10.1021/acsomega.5c12269)
Supplement: Supplementary file 1 [file ao5c12269_si_001.pdf]

Supplementary Information

A Unified Platform for FCS and RICS Analysis with Advanced Statistical Inference

Hamed Karimi[1], Otto Gustavson[1], Irina Česnokova[1], Jelena Branovets[1], Rikke Birkedal[1], Martin Laasmaa[1], and Marko Vendelin*[1]

[1]Laboratory of Systems Biology, Department of Cybernetics, Tallinn University of Technology, Akadeemia tee 15, 12618 Tallinn, Estonia

*Corresponding author: Marko Vendelin. Email: markov@sysbio.ioc.ee

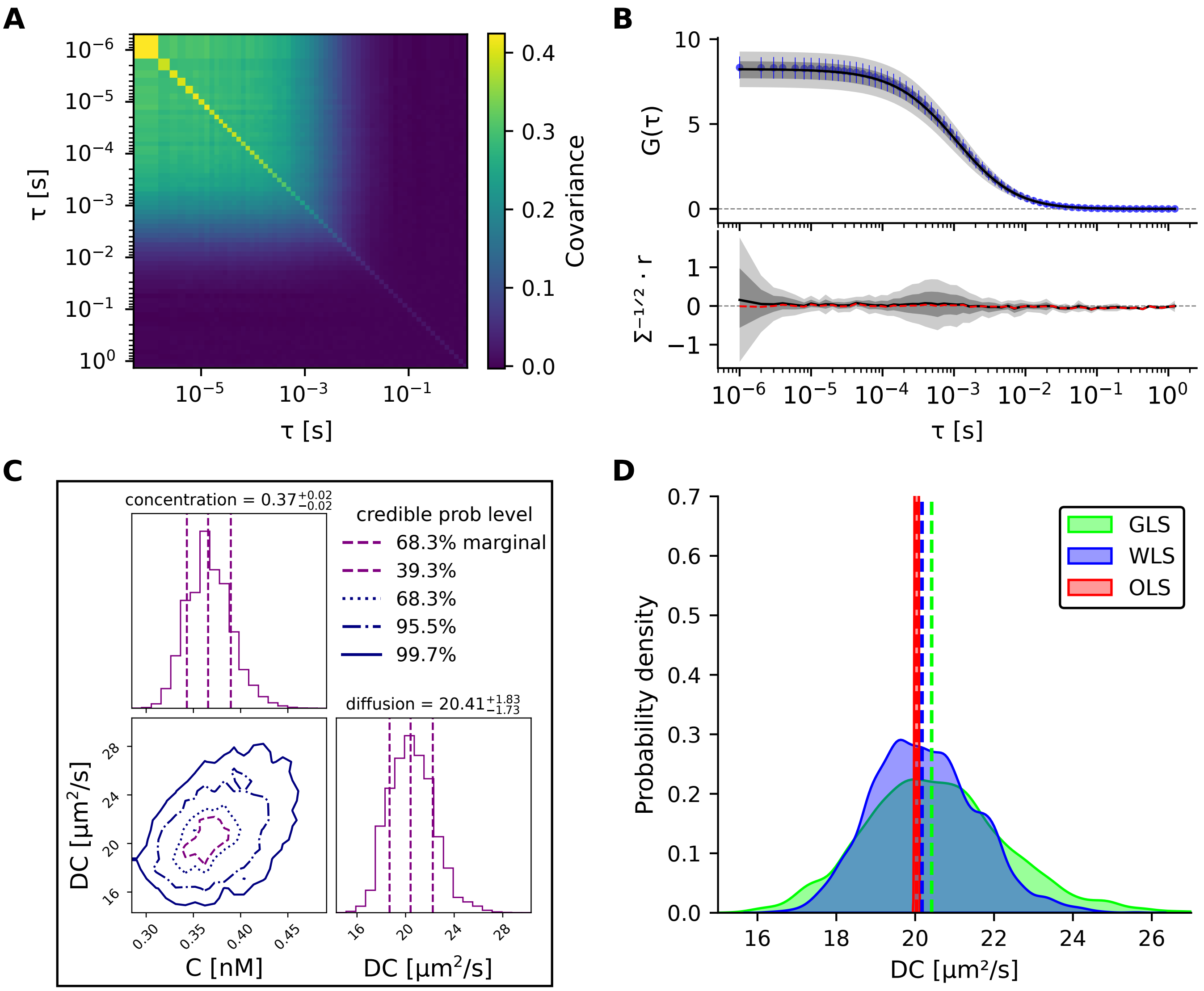

Figure S1: Demonstration of FCS using 2000 synthetic fluorescence traces generated for particles with $DC = 20\ \mu m^2/s$. (**A**) Covariance matrix of the ACF computed from the 2000 individual traces, illustrating the correlation structure of noise across lag times. (**B**) Top: Mean autocorrelation $G(\tau)$ (blue circles) plotted versus τ, along with the median Bayesian GLS fit (black line) based on a 3D isotropic diffusion model. Error bars represent the standard deviation (SD) of the mean. Bottom: Decorrelated residuals calculated from the ACF predictive posterior residual (r) and covariance (Σ), with the black line showing the median residual. The dashed red line indicates the decorrelated residuals from the NLS method (with correlated errors) fit. Shaded regions (top and bottom), shown in dark and light gray, denote the 68.3% and 95.5% QRs, respectively. (**C**) Corner plot showing posterior distributions and pairwise parameter correlations obtained from GLS fitting under Bayesian inference. Values shown above the distributions indicate the median and corresponding 68.3% credible intervals for each parameter. (**D**) Posterior distributions of DC obtained from three fitting approaches: Bayesian GLS (green, correlated errors), Bayesian WLS (blue, weighted errors), and Bayesian OLS (red, uniform errors). Dashed lines indicate the medians of the respective distributions. The OLS posterior distribution (red) is truncated at a probability density of 0.7 in the plot.

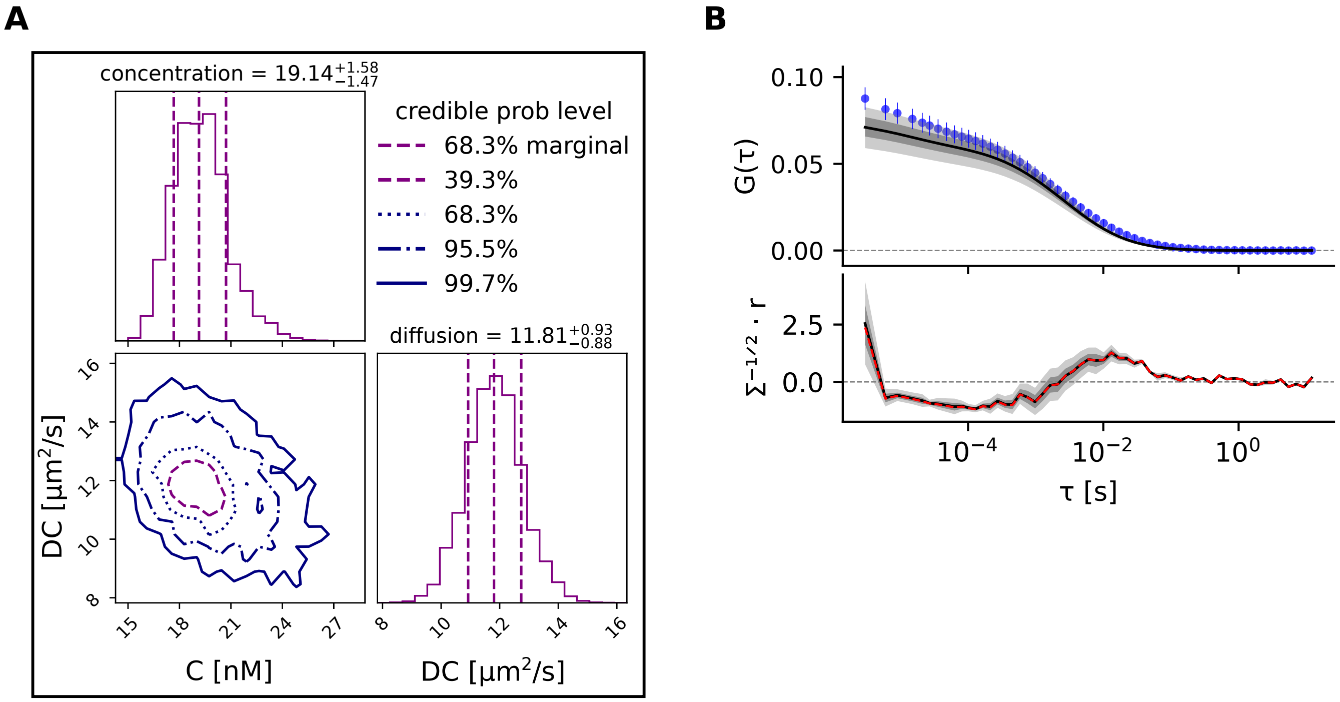

Figure S2: FCS measurements corresponding to Figure 3 in the main text, analyzed using a 3D isotropic diffusion model under Bayesian GLS fitting ($\log Z = 270.02 \pm 0.16$). (**A**) Corner plot showing posterior distributions and pairwise parameter correlations. Values shown above the distributions indicate the median and corresponding 68.3% credible intervals for each parameter. (**B**) Top: Mean autocorrelation $G(\tau)$ (blue circles) plotted versus τ, along with the median Bayesian GLS fit (black line). Error bars represent the SD of the mean. Bottom: Decorrelated residuals calculated from the ACF predictive posterior residual (r) and covariance (Σ), with the black line showing the median residual. The dashed red line indicates the decorrelated residuals from the NLS method (with correlated errors) fit. Shaded regions (top and bottom), shown in dark and light gray, denote the 68.3% and 95.5% QRs, respectively.

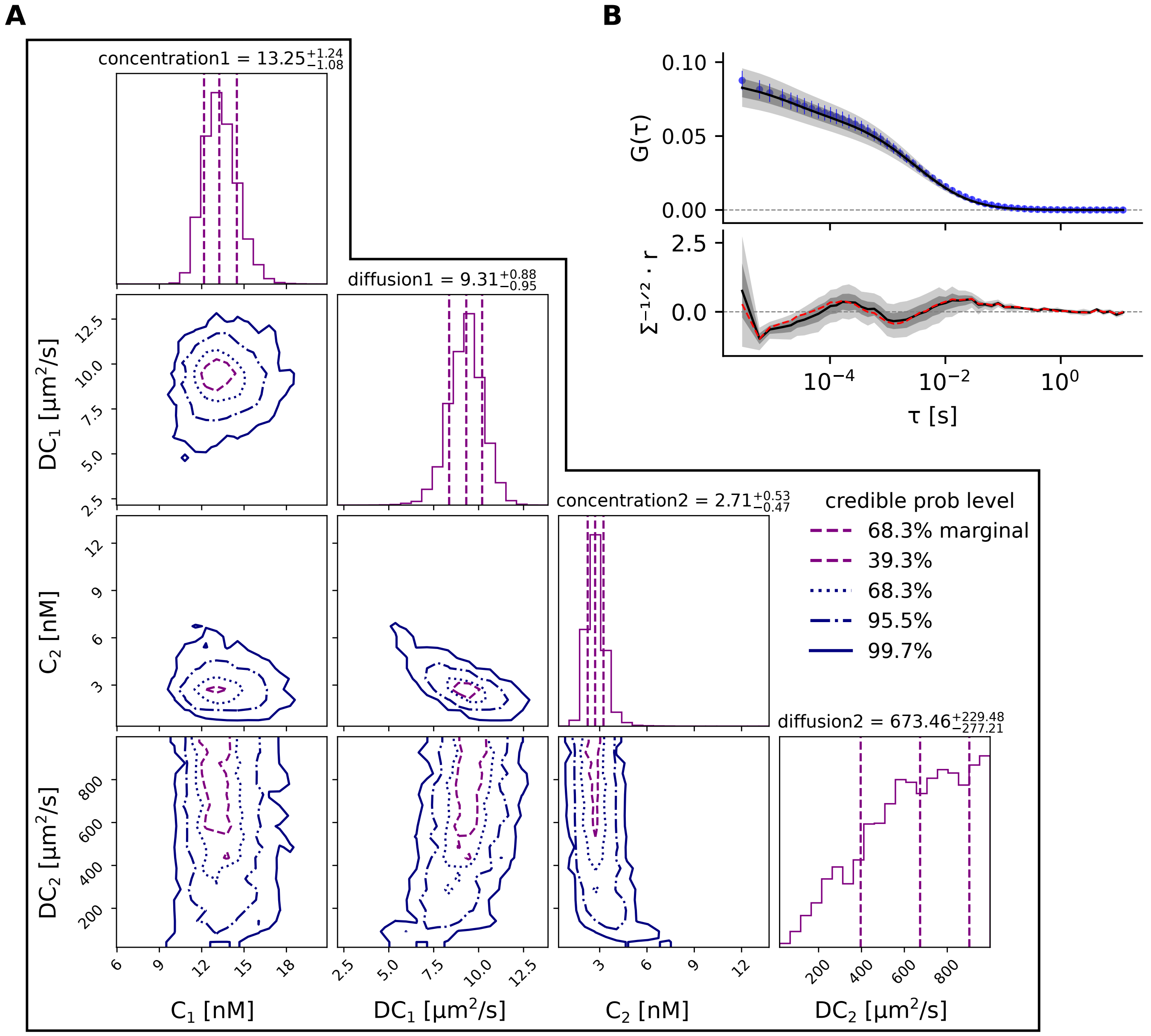

Figure S3: FCS measurements corresponding to Figure 3 in the main text, analyzed using a 3D isotropic two-component diffusion model under Bayesian GLS fitting (log Z = 278.05 ± 0.30). (**A**) Corner plot as described in Figure S2. (**B**) Mean autocorrelation $G(\tau)$ and corresponding Bayesian GLS fit with residuals, as described in Figure S2.

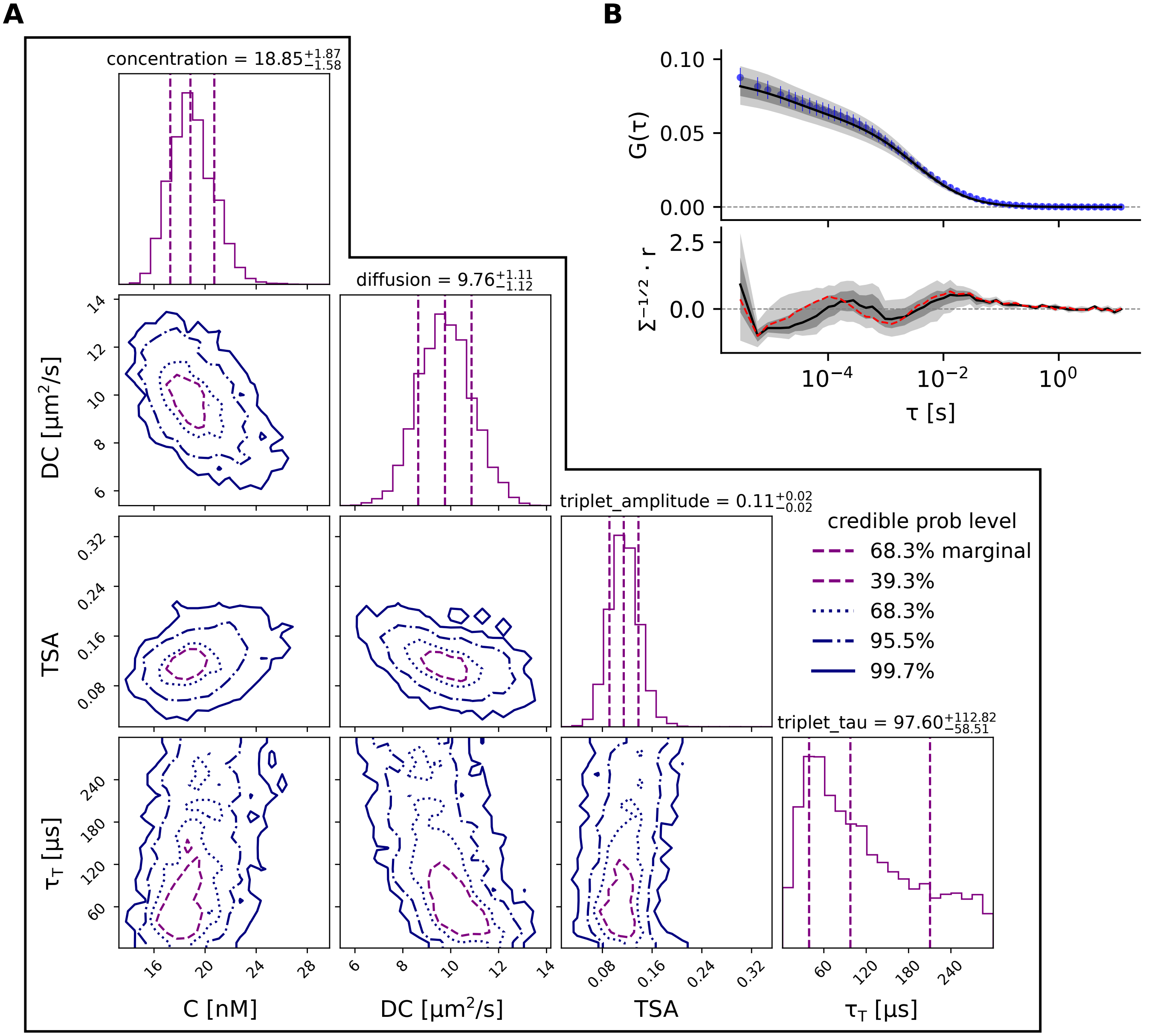

Figure S4: FCS measurements corresponding to Figure 3 in the main text, analyzed using a 3D isotropic diffusion model with an additional triplet-state component under Bayesian GLS fitting ($\log Z = 279.45 \pm 0.23$). The Bayesian evidence confirms that this 3D isotropic diffusion model with a triplet-state component provides the highest evidence score among all models tested. (**A**) Corner plot as described in Figure S2. (**B**) Mean autocorrelation $G(\tau)$ and corresponding Bayesian GLS fit with residuals, as described in Figure S2.

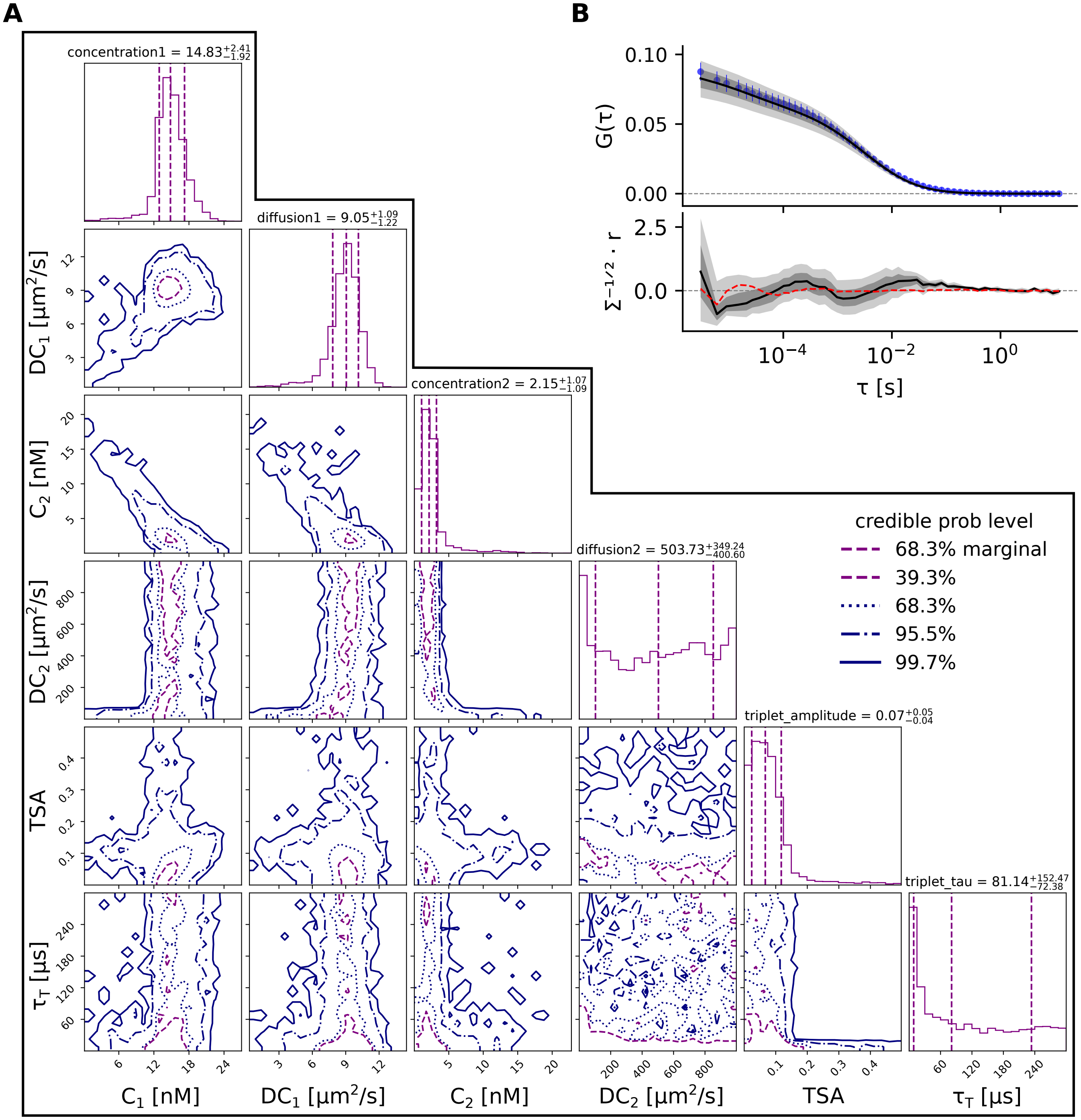

Figure S5: FCS measurements corresponding to Figure 3 in the main text, analyzed using a 3D isotropic two-component diffusion model with an additional triplet-state component under Bayesian GLS fitting ($\log Z = 277.09 \pm 0.23$). (**A**) Corner plot as described in Figure S2. (**B**) Mean autocorrelation $G(\tau)$ and corresponding Bayesian GLS fit with residuals, as described in Figure S2.

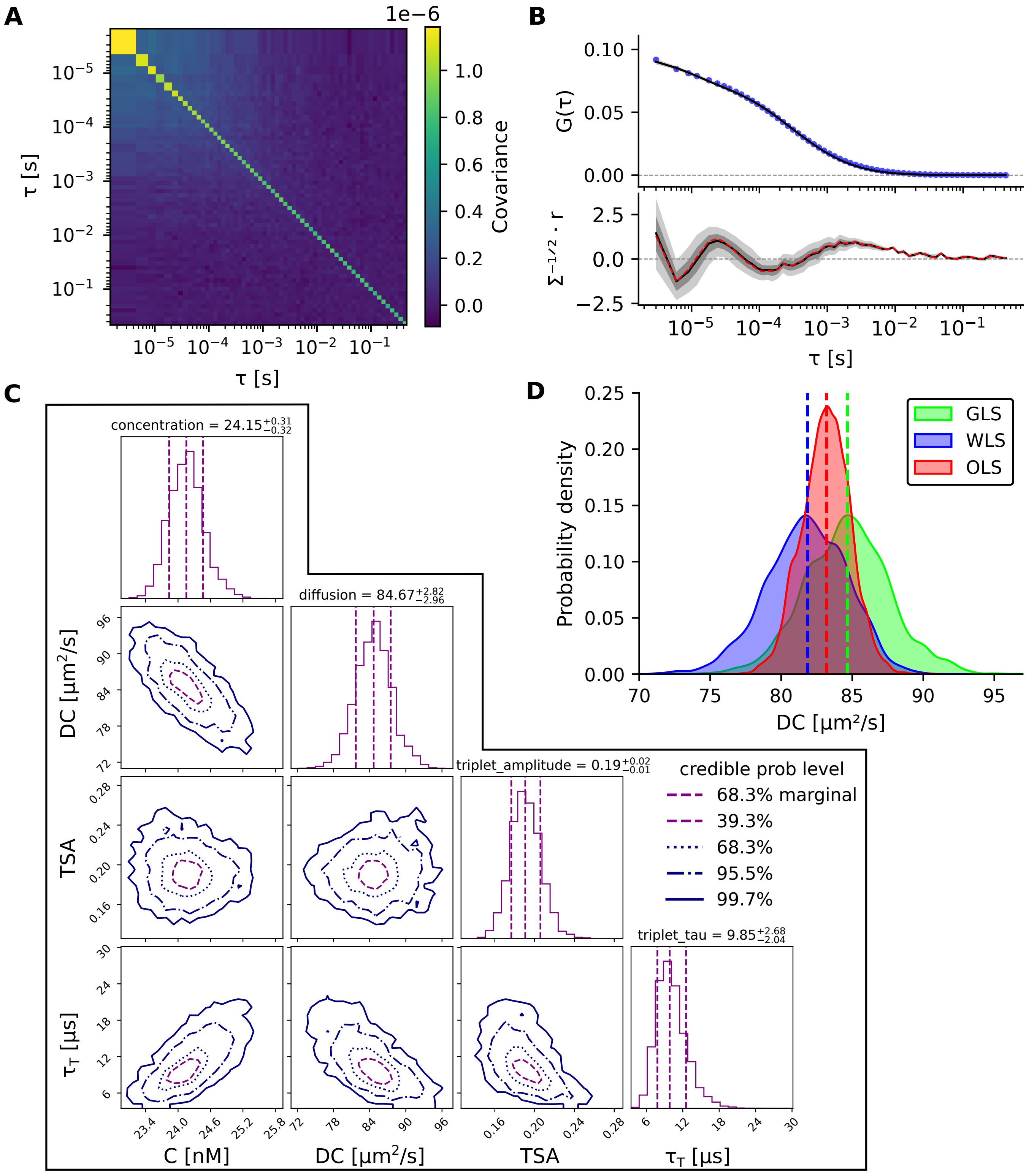

Figure S6: FCS measurements using 1800 experimental traces recorded for diffusion of Alexa Fluor 647-labeled Dextran 10K in water, analyzed using a 3D isotropic diffusion model with an additional triplet-state component. (**A**) Covariance matrix of the ACF computed from the 1800 individual traces, illustrating the correlation structure of noise across lag times. (**B**) Mean autocorrelation $G(\tau)$ and corresponding Bayesian GLS fit with residuals, as described in Figure S1. (**C**) Corner plot as described in Figure S1. (**D**) Posterior distributions of DC obtained from three fitting approaches, as described in Figure S1.
